# Catchment area, fate, and environmental risks investigation of micropollutants in Danish wastewater

**DOI:** 10.1007/s11356-023-30331-z

**Published:** 2023-11-10

**Authors:** Kristoffer Kilpinen, Jason Devers, Mafalda Castro, Selina Tisler, Mathias B. Jørgensen, Peter Mortensen, Jan H. Christensen

**Affiliations:** 1 Department of Plant and Environmental Sciences, University of Copenhagen, Thorvaldsensvej 40, DK-1871 Frederiksberg C, Denmark; 2 Eurofins Environment Denmark, Ladelundvej 85, DK-6600 Vejen, Denmark; 3 Environmental Toxicology, Department of Plant and Environmental Science, University of Copenhagen, Thorvaldsensvej 40, DK-1871 Frederiksberg C, Denmark; 4 BIOFOS a/s Refshalevej 250, DK-1432 Copenhagen, Denmark

**Keywords:** Wastewater epidemiology, Micropollutants, Chemicals of emerging concern, Removal efficiency, Wastewater treatment, Pharmaceuticals, GC × GC-QToF, LC-QToFMS

## Abstract

This study aimed to investigate the spatial distribution of micropollutants in wastewater related to catchment area, and their environmental risks and fate. About 24-h flow proportional effluent (*n* = 26) wastewater samples were collected from eight WWTPs across Denmark. From five of these WWTPs corresponding influent samples (*n* = 20) were collected. Samples were enriched by multi-layer solid phase and analysed by liquid chromatography–high-resolution mass spectrometry and comprehensive two-dimensional gas chromatography with high-resolution mass spectrometry detection. We detected and quantified 79 micropollutants from a list of 291 micropollutants in at least one influent or effluent wastewater sample. From this we found that 54 micropollutants decreased in concentrations during wastewater treatment, while O-desmethylvenlafaxine, carbamazepine, amitriptyline, benzothiazole, terbutryn, and citalopram increased in concentrations through the WWTP.

The toxicity of effluent wastewater samples was assessed by EC50 using *Raphidocelis subcapitata* (*R. subcapitata*) and LC50 using the crustacean *Daphnia magna* (*D. Magna*), for which six micropollutants were detected above the predicted no-effect concentration. Our study demonstrates that catchment area influences the micropollutant composition of wastewater. Out of 19 pharmaceuticals, the measured concentration in influent wastewater was predicted within a factor of 10 from sale numbers and human excretion, which demonstrates the strong influence of catchment area on micropollutant composition.

**Supplementary information:**

The online version contains supplementary material available at 10.1007/s11356-023-30331-z.

## Introduction

Various studies have shown the presence of micropollutants and their transformation products in the environment (Wiest et al. 2021; Rogowska et al. 2020; Gosset et al. 2021; Gago-Ferrero et al. 2020). Insufficient removal of micropollutants in wastewater treatment plants (WWTPs) leads to the discharge of micropollutants into the environment (Rogowska et al. 2020).

Only a few well-known micropollutants are routinely measured in effluent wastewater and the aqueous environment (Gago-Ferrero et al. 2018). Presently, there are no EU regulations in place that require the regular monitoring of pharmaceuticals, industrial chemicals, pesticides and other such micropollutants (Council Directive of 91/5/EC Concerning Urban Waste Water Treatment 1991). The development and improvement of high-resolution mass spectrometers (HRMS) such as time-of-flight (TOF) and Orbitrap mass analysers allow for the development of comprehensive target methods where the sensitivity is not affected by an increasing number of target micropollutants (Gago-Ferrero et al. 2020). However, only a few studies have involved the target screening of > 100 target micropollutants in wastewater and in general, these studies tend to focus on few samples (Gago-Ferrero et al. 2020; Wiest et al. 2021).

A comprehensive target analysis of micropollutants can be achieved by combining liquid chromatography (LC) and gas chromatography (GC), suitable for more polar and non-polar micropollutants, respectively. The combination of methods allows for identifying and quantifying micropollutants from various sources such as industrial chemicals, pharmaceuticals, and pesticides (Moschet et al. 2017).

Understanding the fate of micropollutants in WWTPs is necessary to identify and quantify outflows of micropollutants to the environment. Some micropollutants can be effectively removed in WWTPs, whereas others show little or no removal (Gago-Ferrero et al. 2020; Wiest et al. 2021). Gosset et al. (2021) have shown that a wide range of micropollutants from pharmaceuticals to pesticides, possess environmental risks due to their concentration above the predicted no-effect concentration (PNEC) in effluent wastewater streams (Gosset et al. 2021). To the authors’ knowledge only a few studies have investigated the removal efficiency of > 50 micropollutants across ≥ 5 operational WWTPs (Wiest et al. 2021). Similarly, the prioritisation of micropollutants is often based on PNEC. However, these PNEC values can vary depending on the approach used to estimate them (Gosset et al. 2021; Tranekær et al. 2021), this can make the comparison between studies difficult and result in different prioritisations of micropollutants depending on the referenced PNEC.

Understanding the catchment area for a given WWTP is an essential step towards selecting the right micropollutants for monitoring the release to the environment. One of the most investigated groups of micropollutants in wastewater is pharmaceuticals (Wiest et al. 2021; Rogowska et al. 2020; Gosset et al. 2021; Gago-Ferrero et al. 2020), predominantly discharged from households (Trænekjær et al. 2021). Hospitals prescribed only 3.8–4% of all pharmaceuticals in Denmark in 2015–2019 (Trænekjær et al. 2021). However, it has been shown that for specific pharmaceuticals the contribution from specific sources such as nursing homes and hospitals cannot be neglected (Herrmann et al. 2015). This implies that such sources might affect the inflow to WWTP differently (Herrmann et al. 2015). Similarly, pesticides and biocides might be associated with specific types of farming, green house activities and uses in households; and a wide selection of biocides and pesticides have been detected in wastewater (Köck-Schulmeyer et al. 2013). Industries might be consuming specific micropollutants and each industry can have specific micropollutant compositions (Liu et al. 2022). These micropollutants associated with various industries may influence WWTPs’ chemical composition. However, studies investigating wastewater tend to focus on specific groups of chemicals, such as pesticides (Köck-Schulmeyer et al. 2013), specific pharmaceuticals (Clara et al. 2004; Escolà Casas et al. 2021), household chemicals (Trenholm et al. 2008) or industrial compounds (Liu et al. 2022).

Several studies on wastewater-based epidemiology have shown that wastewater possesses an opportunity to investigate and spot trends in the general public consumption of illicit drugs and pharmaceuticals (P. M. Choi et al. 2019; 2018a, b; Escolà Casas et al. 2021). Concentrations of pharmaceuticals in influent wastewater combined with details on wastewater flow and the number of people connected to a WWTP may make it possible to estimate the amount of a pharmaceutical used in the catchment area (Escolà Casas et al. 2021). One option is to use pharmaceutical prescriptions to do the estimation, but public records are often limited to prescription data (Oosterhuis et al. 2013; Rice et al. 2020; Escolà Casas et al. 2021) or to limited regional data (He et al. 2020). In Denmark, sale numbers of prescriptions for pharmaceuticals from private and public hospitals and pharmaceuticals sold over the counter are publicly available (Sundhedsdatastyrelsen 2021). This allows for a verification of the use of wastewater-based models to back-calculate the consumed amount of pharmaceuticals in a WWTP catchment area.

In this study, we investigated the combination of LC- and GC × GC-HRMS for the target screening analysis of 291 micropollutants in wastewater. Our study aimed to develop and apply the method, to improve our understanding of the fate of micropollutants during wastewater treatment, investigate the influence of the catchment area on the distribution of micropollutants and to assess the risks of released micropollutants.

To investigate the fate of micropollutants in WWTPs, 20 influent and 14 effluent wastewater samples were collected on different days from five different WWTPs across Denmark. For an investigation of the influence of the catchment area on the distribution of micropollutants, a total of 26 effluent wastewater samples from eight different Danish WWTPs with different sizes and catchment areas. The WWTPs range in size from WWTP Otterup, which is scaled for 12,500 population equivalents (PE), to WWTP Lynetten, which is scaled for 750,000 PE. The mass inflow of pharmaceuticals in influent wastewater was compared with the theoretical concentration based on sale numbers and expected excretion of unchanged pharmaceuticals. Risk quotients (*RQ*) were calculated based on calculated PNEC values and measured concentrations to allow for a risk assessment of micropollutants.

## Experimental

### Wastewater treatment plant

Forty-six wastewater samples were collected from eight WWTPs with activated sludge treatment (see Table S1 and Fig. S1). The samples were collected between June 22 and July 21, 2020 (see Table S2). WWTP LY, WWTP AV, WWTP DA, WWTP NE, WWTP NV and WWTP EM are located in Denmark’s largest and third largest cities. Compared, to this WWTP OT and WWTP SO are placed around smaller villages in Otterup with 5229 citizens in 2020 and Søndersø with 3273 citizens in 2020 (Danmmarks Statistik 2021), respectively. For a more detailed description of the WWTPs, see section S1.1.

### Chemicals and reagents

Standard solutions were prepared in MiliQ water, methanol or acetonitrile and stored at − 18 °C (see SI_B, “Standards” for details).

Formic acid (LC-MS grade, > 99% Purity, Fisher Chemical), MiliQ water (Ultrapure, > 18.2 MΩ cm^−1^), and acetonitrile (Chemsolute, purity > 99.95%), LC-MS ultra Chromosolv methanol (Honeywell Riedel-de Haen, Seelze, Germany, purity > 99.9%) were used.

### Sampling and sample preparation

Influent wastewater samples were collected after the bar screen and before or after the grit and grease chamber. Effluent wastewater samples were collected just before the wastewater was discarded into the environment. A total of 20 influent samples from five WWTPs and 26 effluent samples from eight WWTPs were collected for this study (a detailed list of samples is given in Table S2).

About 24-h flow-proportional samples were collected as increments of 100 mL and stored at 4 °C in 10-L glass containers during sampling. Samples were then immediately transported to the laboratory (between 3 and 5 L), pH adjusted to pH 6.5 with formic acid and filtered first through a glass microfiber filter with a diameter of 1.6 µm and subsequently filtered through a filter with a diameter of 0.7 µm. The samples were enriched 500 times (relative enrichment factor of 500, REF 500) using a multilayer-solid phase extraction (ml-SPE) method published by Tisler et al. (2021). A short description of the ml-SPE method is given in S1.4. Internal standards were added to the ml-SPE extracts and standard solutions prior to analysis (see section S1.4.1 for more details).

### Instrumental analysis, quantification and quality assurance

A list of 291 target micropollutants were analysed by LC-HRMS using an Acquity Ultra-Performance Liquid Chromatograph (UPLC) equipped with a Synapt G2S quadrupole time of flight mass spectrometry (QTOFMS) (Waters, Taastrup, Denmark). The GC × GC-HRMS analysis was performed using an Agilent 7890B GC system equipped with an Agilent 7200 Accurate Mass QTOFMS (Agilent Technology, Palo Alto, CA, USA). The first column was a 60-m, 0.25-mm id, 0.25-µm non-polar ZB-5 column (Phenomenex, Torrance, CA, USA), and the secondary column was 1.5-m, 0.18-mm i.d., 0.18-µm mid-polar ZB-50 column (Phenomenex, Torrance, CA, USA). The modulator was a Zoex ZX2 cryogen-free modulator (Zoex Corporation, Houston, TX, USA). For a detailed description of the LC-QTOF and GC × GC-QTOF method, see sections S1.2.1 and S1.2.2, respectively.

For both LC-QTOF and GC × GC-QTOF, concentrations were quantified using internal standard calibration. A detailed description on quality assurance is described in section S1.5.

### Risk quotient calculation

For estimation of the environmental risk of the quantified micropollutants, *RQ* has been calculated according to Eq. (1). *RQ* has only been calculated for compounds with at least one detection above the LOQ in effluent wastewater.

1 $${\varvec{R}}{\varvec{Q}}=\frac{{\varvec{M}}{\varvec{E}}{\varvec{C}}}{{\varvec{P}}{\varvec{N}}{\varvec{E}}{\varvec{C}}}$$

where *MEC* is the measured wastewater concentration; and PNEC (predicted no effect concentration) is the EC_50_ value from the acute test using the freshwater species *Daphnia magna* (*D. magna*) (OECD 2004) or growth inhibition test using *Raphidocelis subcapitata* (*R. subcapitata*) (OECD 2011) divided by an assessment factor of 1000, calculated according to the EU Commission Directive EUR 20418 EN/2 (De Bruijn et al., 2003). Experimental EC_50_ values for *R. subcapitata* and *D. magna* were collected from the ECOTOX Knowledgebase (Environmental Protection Agency 2022) (SI_B, “DMagna_Tox” and “Rsubcapitata_Tox”). All EC_50_ values that complied with the OECD guidelines (i.e. age of organisms at the start of the experiment and duration of the experiment) were used in this study.

A Monte Carlo simulation was made to include all toxicological tests in the study, combining the different MEC and PNEC values. The assumption was made that all test results had the same likelihood of being correct. Data was transferred to Excel (Microsoft, Redmond, USA) where the Monte Carlo Simulation was made using the built-in random function (0 to 1). One hundred guesses were made, where 100 PNEC values for each micropollutant were simulated (within low and max PNEC); these were then combined with the MEC, and the data was transferred to R (R Core Team 2018), and a boxplot was constructed.

### Hierarchical clustering and boxplot

The data was transferred to R (R Core Team 2018, version 4.0.3); each micropollutant was range-scaled, where the highest concentration was set at one, and the lowest value was set at zero. Boxplot with hierarchical clustering was constructed using the library pheatmap (version 1.0.12). The clustering was done using the build-in clustering method: complete-linkage clustering.

Pearson correlation coefficients were calculated in R (R Core Team 2018), using the build-in correlation method: Cor (Statistical Package, included in R Version 4.0.3).

### Fate of micropollutants in WWTP

The effluent wastewater concentration was compared to the influent concentrations according to Eq. (2).

2 $${\varvec{E}}{\varvec{f}}{\varvec{f}}{\varvec{l}}{\varvec{u}}{\varvec{e}}{\varvec{n}}{\varvec{t}}\boldsymbol{ }{\varvec{c}}{\varvec{o}}{\varvec{m}}{\varvec{p}}{\varvec{a}}{\varvec{r}}{\varvec{e}}{\varvec{d}}\boldsymbol{ }{\varvec{t}}{\varvec{o}}\boldsymbol{ }{\varvec{i}}{\varvec{n}}{\varvec{f}}{\varvec{l}}{\varvec{u}}{\varvec{e}}{\varvec{n}}{\varvec{t}}=(1-\frac{{{\varvec{C}}{\varvec{o}}{\varvec{n}}{\varvec{c}}}_{\mathbf{I}\mathbf{n}\mathbf{f}}-{{\varvec{C}}{\varvec{o}}{\varvec{n}}{\varvec{c}}}_{\mathbf{e}\mathbf{f}\mathbf{f}}}{{{\varvec{C}}{\varvec{o}}{\varvec{n}}{\varvec{c}}}_{\mathbf{I}\mathbf{n}\mathbf{f}}})\bullet 100\mathbf{\%}$$

where, *Conc*_Inf_ is the concentration of the micropollutant in the influent sample, and *Conc*_eff_ is the concentration of the micropollutant in the effluent sample. The effluent compared to influent was calculated for days with a 24-h composite sample on the same day for both influent and effluent wastewaters from the same WWTP, and where micropollutant concentrations were above the LOQ in both the influent and effluent sample. For cases where a micropollutant was measured in the influent samples but not in the effluent sample, the effluent compared to influent was set to 0%.

### Consumed pharmaceuticals

For the investigated pharmaceuticals, Anatomical Therapeutic Chemical codes were obtained from the World Health Organization (WHO Collaborating Centre for Drug 2021). The total sale amount of defined daily dose (DDD) for 1000 people per day was obtained for each pharmaceutical on a yearly and regional scale using data from the Danish Medicines Agency (Sundhedsdatastyrelsen 2021). This data included both pharmaceuticals sold on prescriptions and over the counter. The DDD was then calculated into a concentration using information about the amount of active ingredient obtained from the World Health Organization (WHO Collaborating Centre for Drug 2021). Pharmaceuticals were only included in this study if it was possible to obtain all the information mentioned above (Table S5).

A recently suggested workflow used the excretion of each pharmaceutical in urine and faeces (Escolà Casas et al. 2021). A combined excretion (%) of pharmaceuticals was obtained from the work of Escolà Casas et al. (2021)or, in a few cases, from other literature sources. A value was obtained for the lowest expected excretion and the highest expected excretion (Table S5). Theoretical concentrations were then calculated using Eq. (S2), taking into account the lowest and highest expected excretion values presented in Table S5. Measured concentrations were determined using the wastewater flow and the population connected to the WWTP, as outlined in Eq. S3.

## Results and discussion

### Concentrations and detection frequencies of micropollutants in wastewater

Quantification limits were determined for all 291 investigated micropollutants and are presented in SI_B, “Detected_Compounds” and “Non-detected_Compounds.”. For the micropollutants detected in at least one sample, linearity was investigated (SI_B, “Detected_Compounds”). Matrix effects for these samples have previously been investigated and published (Tisler et al. 2021). For 67 micropollutants, the average matrix effects were determined to be − 19% (standard deviation: 18%) in effluent wastewater at REF 50 and − 16% (standard deviation: 24%) in influent wastewater at REF 10 (Tisler et al. 2021). Recoveries were assessed for a representative subset of micropollutants, consisting of 27 compounds, as detailed in Table S6. The recoveries ranged from 14 to 189% in effluent wastewater, with the first quartile at 71% and the third quartile at 93% (see Table S6).

**Fig. 1:**
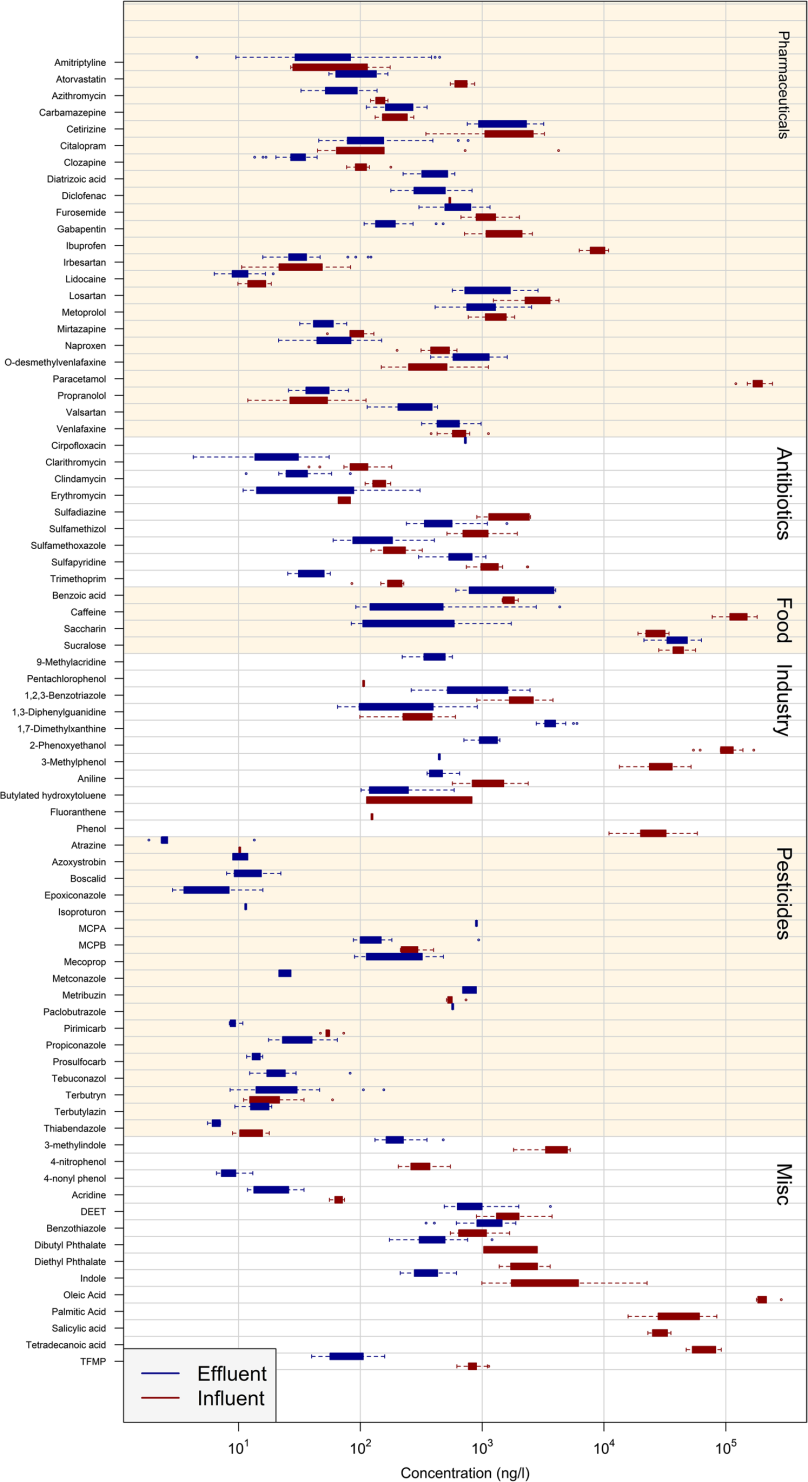
Concentrations (ng L^−1^) of all micropollutants quantified in this study (detected in at least one sample). The results are shown for each micropollutant for effluent wastewater (blue) and influent wastewater (red). The micropollutants are separated into groups based on primary use: pharmaceuticals, antibiotics, pesticides, food additives and compounds that do not fit into other categories (Misc.)

**Fig. 2:**
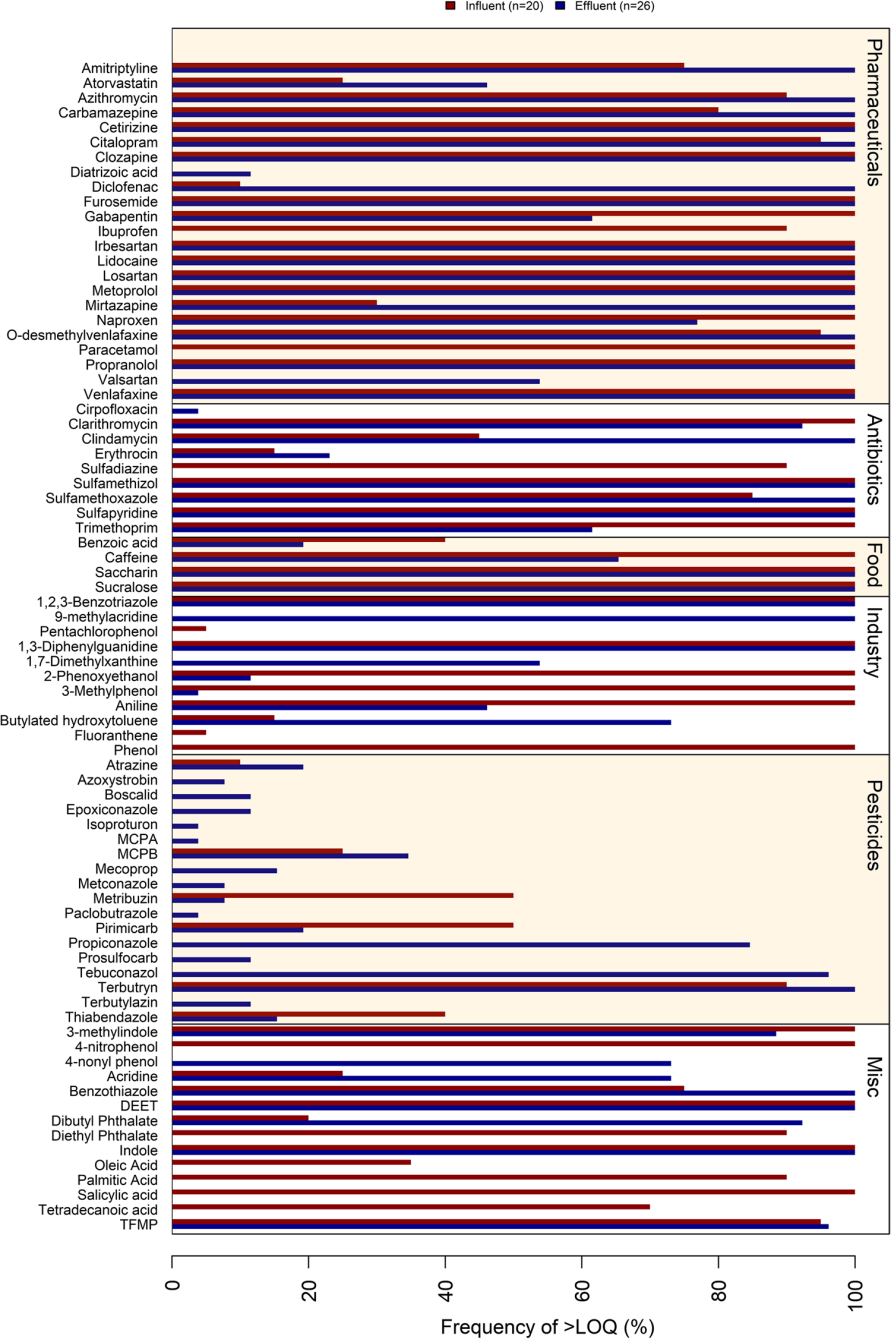
The quantification frequency of the target micropollutants. This study included 20 influent wastewater samples from five WWTPs (red) and 26 effluent wastewater samples from eight WWTPs (blue). The micropollutants are separated into groups based on primary use: pharmaceuticals, antibiotics, pesticides, food additives and compounds that do not fit into other categories (Misc.)

Seventy-nine micropollutants out of the 291 micropollutants monitored in this study were identified and quantified in influent or effluent wastewater from at least one WWTP (SI_B, “Results”). Sixty-one and 67 micropollutants were quantified for influent and effluent wastewater, respectively (Figs. 1 and 2). Twenty-one pharmaceuticals were quantified in at least one influent or effluent sample; 12 pharmaceuticals were quantified in all influent wastewaters, and 16 pharmaceuticals were quantified in all effluent wastewaters. Furthermore, eight antibiotics, four food additives and eight industrial chemicals were quantified in both influent and effluent wastewater; while eight industrial chemicals were quantified only in effluent wastewater and nine only in influent wastewaters. Eighteen pesticides were quantified in effluent wastewaters, while six pesticides were only found in influent wastewaters. Finally, eight micropollutants of mixed origin were found in effluent wastewater, and 13 micropollutants of diverse origin were found in influent wastewater.

The detection frequencies in this study are biased due to the different numbers of influent (*n* = 20) and effluent (*n* = 26) wastewater samples analysed. Influent samples were collected from five WWTPs, while effluent samples were collected from eight WWTPs. Additionally, effluent samples were analysed at a higher enrichment factor (REF 50) compared to influent samples (REF 10) to minimise matrix effects.

Paracetamol, caffeine, 2-phenoxyethanol and oleic acid were measured in the highest concentrations across all wastewater samples, all four micropollutants with influent concentrations above 100 µg L^−1^. For these high concentration micropollutants, the difference in concentrations between WWTPs was highest for caffeine, with a factor of 2.3 between the highest and lowest concentrations in influent wastewater across WWTPs. The relatively low variation between WWTPs indicates that these chemicals do not have specific point sources. For all of these four micropollutants, high influent wastewater concentrations of above 25 µg L^−1^ has previously been reported (Gómez et al. 2007; Kjølholt and Schmidt, 2021; Trenholm et al. 2008; Haussard et al. 2002).

Paracetamol is a painkiller previously reported in high concentrations in influent wastewater. 2-Phenoxyethanol has multiple uses in industry and as an additive to cosmetics (Trenholm et al. 2008). Oleic acid has many origins. The body naturally produces it, and it is found in many types of food, and it is known to be disposed to wastewater from commercial kitchens (Haussard et al. 2002). Oleic acid was quantified in concentration range of 180 to 290 µg L^−1^ with a detection frequency of 35% in influent wastewater (LOQ for oleic acid: 172 µg L^−1^). Neither paracetamol nor oleic acid was detected > LOQ in effluent wastewater. Caffeine was found > LOQ in 65% of the effluent samples in concentrations ranging from 0.09 to 4.3 µg L^−1^ and 2-phenoxyethanol was found > LOQ in 12% of the effluent samples in concentrations ranging from 0.7 to 1.4 µg L^−1^.

**Fig. 3:**
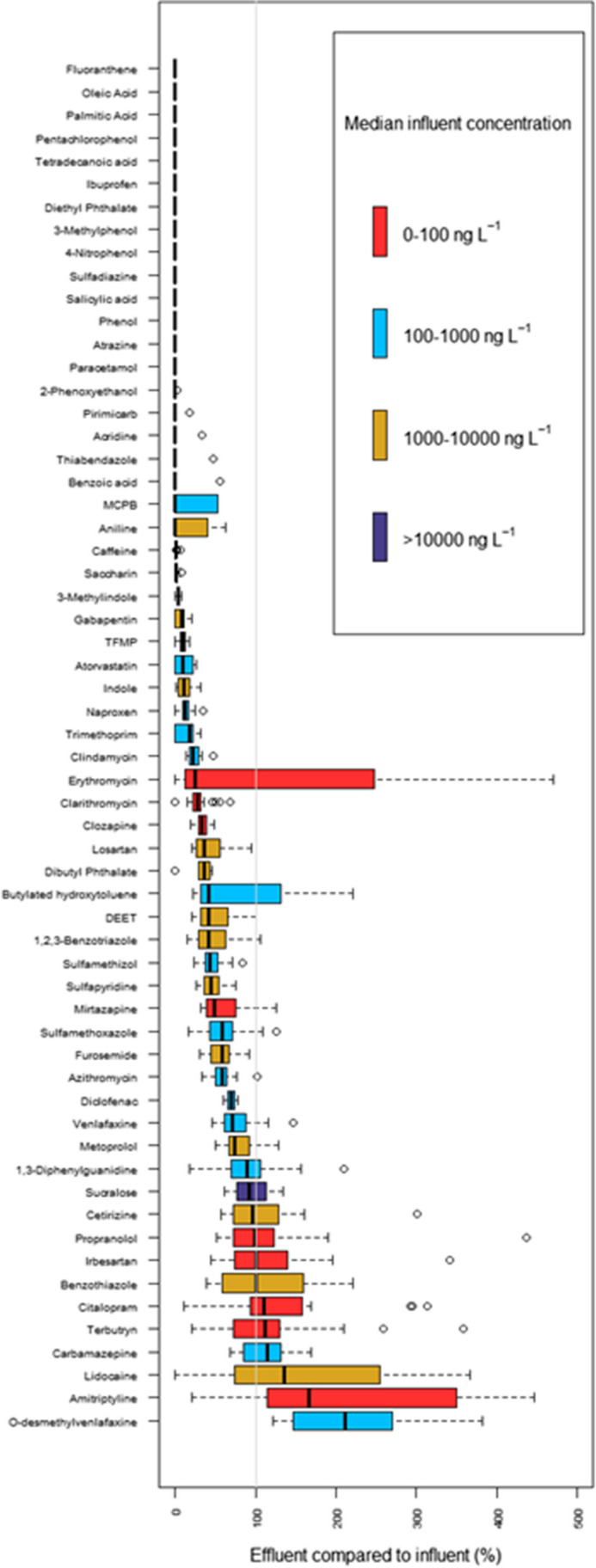
Effluent compared to influent wastewater concentrations. Median influent concentration ranges: 10–100 ng L^−1^ (red), 100–1000 ng L^−1^ (light blue), 1000–10,000 ng L^−1^ (golden) and > 10,000 ng L^−1^ (dark blue). Values < 100% indicates removal through the WWTP, whereas values > 100% indicates formation during WWTP

For effluent wastewater, the highest concentration was observed for the artificial sweetener sucralose with a concentration range of 21.3 to 63.1 µg L^−1^. Previous studies have shown a removal efficiency of 8.6% for sucralose in wastewater (Kadokami et al. 2021). Subsequently, other studies have found high effluent concentrations of sucralose in the range 2 to 55 µg L^−1^ (Subedi and Kannan 2014; Gago-Ferrero et al. 2020; Kadokami et al. 2021). Of the 11 micropollutants with at least one influent measurement above 10 µg L^−1^, only sucralose was measured > 5 µg L^−1^ in effluent wastewater (Fig. 3). Thus, the WWTPs show high efficiency in removing the high-concentration micropollutants with influent concentration > 10 µg L^−1^.

All pesticides found above LOQ were found in quantities < 1 µg L^−1^. Of the 18 pesticides quantified in effluent wastewater, terbutryn, propiconazole and tebuconazole were the most frequently detected pesticides with a detection frequency of ≥ 85%. Terbutryn, propiconazole and tebuconazole were measured in concentrations between 9 and 156 ng L^−1^ for each micropollutant in effluent wastewater. All three have been previously detected multiple times in wastewater in concentrations in the range 0.4 to 115 ng L^−1^ (Hansen et al. 2021; Gago-Ferrero et al. 2020; Wiest et al. 2021; Bollmann et al. 2014). Of the 18 pesticides, 14 were detected in less than 20% of the effluent wastewater samples. Azoxystrobin, metconazole and paclobutrazole were only quantified at WWTP OT, which suggests a local source. Of the investigated WWTPs, WWTP OT and WWTP SO were the only WWTPs located outside large cities. For both WWTP OT and WWTP SO, only effluent wastewater was included.

### Fate of micropollutants in WWTPs

The effluent and influent concentrations were compared according to Eq. (2) (Fig. 3). Fifty-four micropollutants had median concentrations in effluents compared to influent < 100%, meaning that they were removed to some degree through the treatment (Fig. 3). Of these, 14 micropollutants were removed to a degree so that they could only be detected in influent and not effluent wastewater. O-desmethylvenlafaxine, carbamazepine, amitriptyline, benzothiazole, terbutryn and citalopram were found at higher median concentrations in effluent compared to influent wastewater. The reason for this increase in concentration through the WWTP may be manifold: For O-desmethylvenlafaxine, it can be explained by transformation of venlafaxine to O-desmethylvenlafaxine during microbiological treatment (Gasser et al. 2012). Carbamazepine has been shown to enter WWTPs in conjugated forms. The conjugated forms are then de-conjugated during biological treatment (Archer et al. 2017). Thus, resulting in an increase in the concentration of carbamazepine in the effluent wastewater, an increase in effluent compared to influent concentrations can also be due to the micropollutants entering as accumulated aggregates that are separated during the biological treatment (Archer et al. 2017). Amitriptyline has been shown to be persistent in WWTPs (Lajeunesse et al. 2008). Lajeunesse et al. (2008) found amitriptyline concentrations in influent of 17.6 to 20.8 ng L^−1^ compared to 15.6 to 21 ng L^−1^ in effluent wastewater. The main removal pathway of amitriptyline has been suggested to be sorption to particles (Choi et al. 2018a, b). The concentration of amitriptyline in effluent wastewater was higher at WWTP DA compared to the other seven WWTPs (further details in 3.4). The median value of effluent concentration compared to influent for amitriptyline was 166%, whereas for the five WWTP AV measurements, the percentage was 255 to 3077%. Amitriptyline is mainly excreted in glucuronide forms. Only 2% of the initial dose is excreted as amitriptyline (ASHP 2020). It can be speculated that these glucuronide forms are transformed back to amitriptyline by microorganisms resulting in higher concentrations in effluent wastewater at WWTP AV, as has been shown for venlafaxine and carbamazepine (Archer et al. 2017).

An increase in the concentration from influent to effluent wastewater was also observed for terbutryn. Previous studies have shown a similar trend with a low mean removal of terbutryn in the range − 6 to 24% (Wiest et al. 2021; Campo et al. 2013). For the anti-depressive pharmaceutical citalopram, removal efficiencies of 2.3% has previously been shown (Ivanová et al. 2017). However, other studies have shown some reduction in the range 35 to 100% with a mean removal efficiency of 79% (Silva et al. 2014). For benzothiazole, other studies have shown removal efficiencies above 64% (Asimakopoulos et al. 2013).

Six micropollutants showed remaining median concentration between 80 and 99%; 15 micropollutants showed remaining concentrations between 30- and 80%, and 32 micropollutants show median remaining concentrations of < 30%.

A comparison of concentrations was made for samples where an influent and effluent sample was collected on the same day. Sampling was done as 24-h composite sampling. The hydraulic retention time of wastewater in the WWTP was not considered. The total wastewater hydraulic retention time in the WWTPs fluctuated from 27 to 71 h (Table S2). Therefore, there will be differences in the water bodies measured as influent and effluent wastewater, which can explain some variances. It has previously been suggested to use sampling on consecutive days for comparison of effluent and influent wastewater (Ort et al. 2010). Ejhed et al. (2018) showed the difficulties in selecting a sampling strategy for comparison of effluent and influent wastewater. They spiked lithium to influent wastewater and followed it through three WWTPs. The maximum lithium concentration was measured in effluent wastewater after 3, 2 and 3 days, for WWTPs with a calculated hydraulic retention of 3.6, 4.0 and 4.2 days, respectively (Ejhed et al. 2018). Ejhed et al. (2018) also showed that it took 16 days from the spike of the influent wastewater before the concentration of lithium was back to background levels, with the highest concentrations within the first 8 days. Overall, the mixing of the wastewater in the WWTP makes it difficult to sample the same influent and effluent water package, and this was a limitation to this study.

In our study, we did not investigate the specific mechanisms responsible for the differences in concentrations between influent and effluent wastewater, such as adsorption to particles and microbiological degradation. However, our findings indicate that these observed differences in concentrations between influent and effluent wastewater are predominantly determined by the nature of the micropollutants themselves rather than the specific WWTP. Thus, the relative variations in concentrations between influent and effluent wastewater appear to be more micropollutant specific rather than dependent on the WWTPs. However, for the 14 micropollutants that were only measured in influent wastewater, this takes place at all WWTPs. Similarly, for the 24 micropollutants with the highest effluent compared to influent wastewater concentrations, no WWTP is shown to outperform others. For example, for amitriptyline, one measurement out of six from WWTP LY and one out of two measurements from WWTP NE show effluent compared to influent concentrations below < 40%; all the other measurements show effluent compared to influent concentrations above 80%. Similar, trends can be found for the other micropollutants (see Fig. S5 for more details). The average effluent compared to influent concentrations of all micropollutants were 39%, 52%, 62%, 58%, and 46% for WWTP LY, WWTP, AV, WWTP DA, WWTP EM, and WWTP NE, respectively.

### Environmental risk assessment of micropollutants in effluent wastewater

**Fig. 4:**
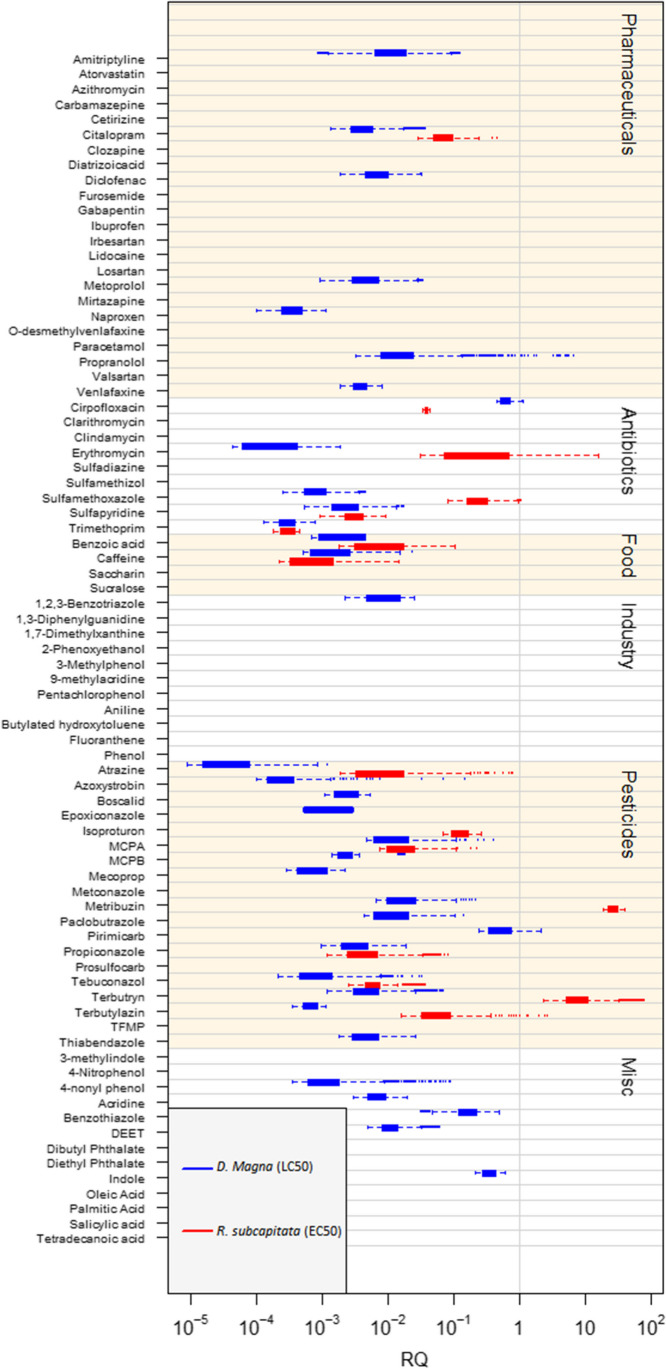
*RQ* is calculated for each quantified micropollutant based on Eq. (1). *RQ* is calculated for *D. Magna* (blue) and *R. subcapitata* (red) based on LC50 and EC50 values, respectively. The micropollutants are separated into groups based on primary use: pharmaceuticals, antibiotics, pesticides, food additives and compounds that do not fit into other categories (Misc.)

To evaluate the environmental risk of the quantified micropollutants, *RQ* was calculated according to Eq. (1) and can be seen in Fig. 4. Eight micropollutants were found to have at least one *RQ* above 1. Propranolol, ciprofloxacin and pirimicarb were found to have *RQ*s > 1 for *D. magna*, whereas erythromycin, sulfamethoxazole, metribuzin, terbutryn and terbuthylazine were found to have *RQ*s > 1 for *R. subcapitata*.

Propranolol, a pharmaceutical used to treat high blood pressure (Medicin.dk 2019), was quantified in all effluent samples. On the other hand, pirimicarb, an insecticide was only quantified in the wastewater of the Funen-based WWTPs. Most products containing pirimicarb have been banned from use in Denmark since December 2020 (SEGES 2021). The antibiotic ciprofloxacin was quantified in one effluent wastewater sample (AV0629, from WWTP AV).

Sulfamethoxazole and erythromycin are antibiotics that were found to have *RQ* values for *R. subcapitata* in the range of 0.08 to 1.1 and 0.03 to 16, respectively. Sulfamethoxazole was detected in all effluent wastewater samples, whereas erythromycin was detected in seven samples spread across the tested WWTPs. *RQ*s above one was previously detected in both surface and wastewater for sulfamethoxazole and erythromycin (Zhang et al. 2021). Metribuzin was only detected in the two effluent wastewater samples from WWTP OT, but in these wastewater samples, it achieved *RQ* values from 19 to 40. Metribuzin is a herbicide used to produce tomatoes and potatoes (SEGES 2021) but has been restricted in Denmark since 2011 (SEGES 2021). Terbuthylazine, another herbicide, was detected in three effluent wastewater samples, of which two samples were from WWTP OT. The *RQ* of terbuthylazine was in the range of 0.01 to 2.7. Lastly, terbutryn was quantified in all effluent wastewater samples and had *RQ* values for *R. subcapitata* between 2.4 and 81. Terbutryn has the primary usage as a herbicide to control grasses and weeds, it is also used as an algicide in textiles production and building materials as a biocide (Vollertsen et al 2017). *RQ* factors above 1 for terbutryn in effluent wastewater have been previously described for French wastewater (Gosset et al. 2021).

In previous studies, even though the environmental concentrations found for venlafaxine were in a range comparable to the ones found in this study (i.e. in the range of 0.01 to 0.77 µg L^−1^, compared to the Venlafaxine concentration range of 0.32 to 0.97 µg L^−1^ reported in this study, (Tranekær et al 2021), the PNEC value used to calculate the *RQ* varied by multiple orders of magnitude. In this study, the lowest PNEC value for venlafaxine is 116 µg L^−1^ (based on the EC_50_ value found for *D. magna*). However, Tranekær and colleagues used a PNEC of 0.1 µg L^−1^ (Tranekær et al 2021) and Gosset and colleagues a PNEC value of 0.000313 µg L^−1^ (Gosset et al. 2021). For Gosset et al. (2021), this lower PNEC value was due to the toxicity of venlafaxine toward freshwater snails (Fong and Hoy 2012). This means that, even though similar venlafaxine concentrations are measured, the final risk assessment provides very different results, depending on which species the toxicity is considered.

Calculating *RQ* is a widely used quotient used to summarise the risk level of a specific chemical. However, it has multiple limitations. *RQ*s are based on environmental concentrations and experimental toxicity values, which are both associated with uncertainties that can lead to an over- or underestimation of the risk level. Moreover, ecotoxicological data is not available for every chemical quantified in the wastewater samples, limiting the *RQ*’s calculation. This was the case for 36 of the quantified micropollutants in this study. Either this is due to a lack of experiments or inactivity of the compound (e.g. EC50 above water solubility). The combination of multiple experimental EC_50_ values can therefore derive a range of *RQ* instead of a single value allowing us to prioritise the micropollutants of concern. Nevertheless, micropollutants with *RQ* < 1 cannot necessarily be considered environmentally safe. Physical-chemical properties that affect environmental fate, such as mobility, environmental persistence and bioaccumulation are also important to consider during environmental risk assessment. The model also does not take the dilution of wastewater in the environment into consideration. There will most often be a significant dilution of the wastewater when it is released into the environment (Gosset et al. 2021). Lastly, this model only considers the toxicity of *D. Magna* and *R. subcapitata*, and other possible non-target organisms are not considered.

### Investigation of differences in micropollutant composition between WWTPs

**Fig. 5:**
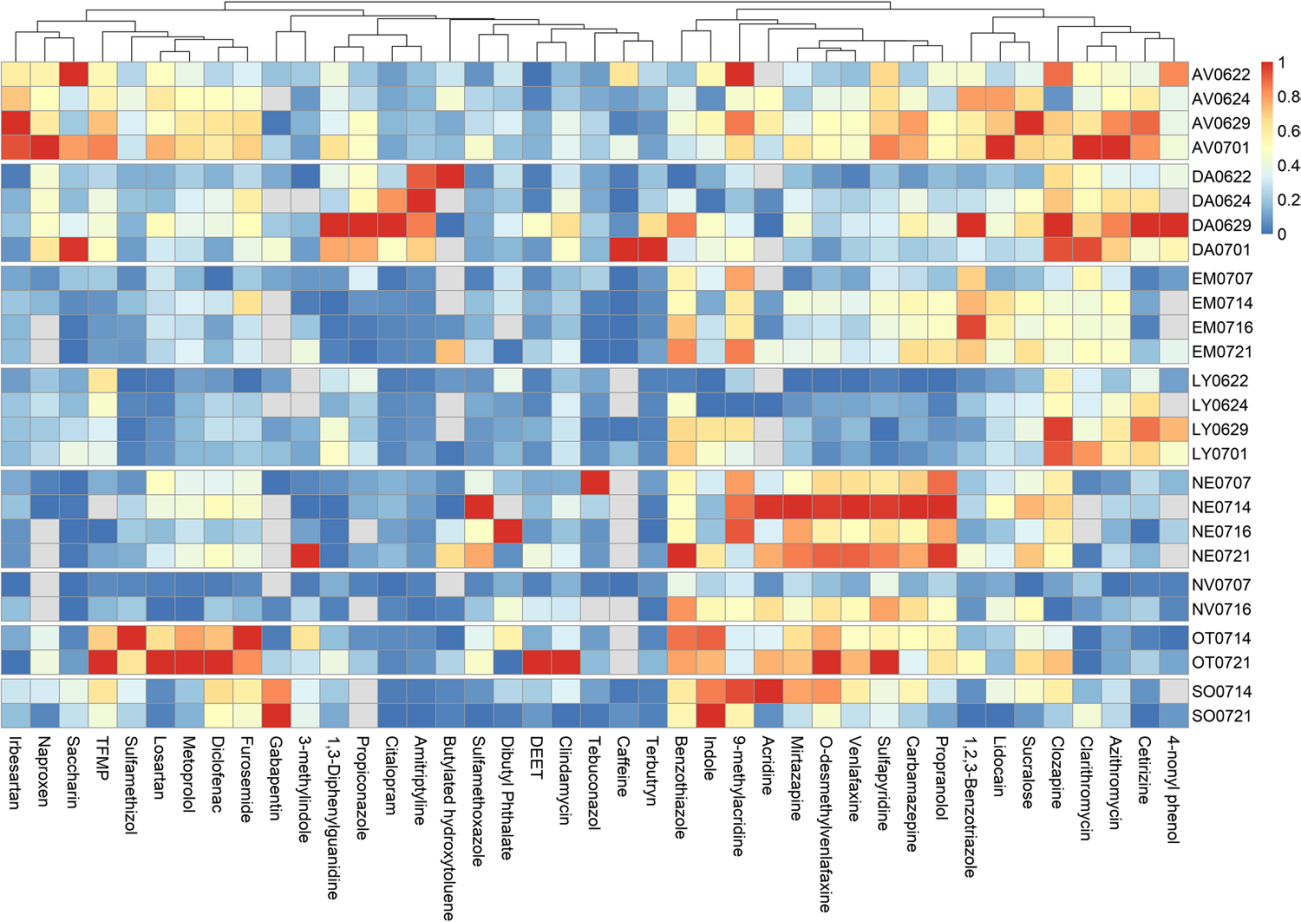
Heat map including hierarchical clustering of effluent wastewaters for all micropollutants (with more than 18 quantifications). For each micropollutant the concentration has been range scaled (lowest value = 0 and highest value = 1)

The range scaled distribution of the most abundant micropollutants (*n* > 18) in effluent wastewater is shown in Fig. 5 (for influent wastewater, see Fig. S6). Covariance between sample concentrations was observed for furosemide, diclofenac, metoprolol and losartan (see Fig. 5). Furosemide, losartan and metoprolol are used to treat high blood pressure, whereas diclofenac is used to treat osteoarthritis and rheumatoid arthritis (Wishart et al. 2018). Metoprolol was found with an average concentration in effluent wastewater across all WWTPs of 1.12 µg L^−1^. The average concentration of metoprolol in both WWTP AV and WWTP OT was 1.57 µg L^−1^. In effluent wastewater, losartan was found at an average concentration of 1.27 µg L^−1^, 1.92 µg L^−1^ and 2.32 µg L^−1^ across all WWTPS, at WWTP AV and WWTP OT, respectively. Furosemide was found at an average concentration of 0.65 µg/L across all WWTPs, 0.77 µg L^−1^ at WWTP AV, 1.09 µg L^−1^ at WWTP OT, and 0.75 µg L^−1^ at WWTP SO and Diclofenac was found at an average concentration of: 0.40 µg L^−1^ across all WWTPs, 0.50 µg L^−1^ at WWTP AV, 0.74 µg L^−1^ at WWTP OT and 0.55 µg L^−1^ at WWTP SO. These findings are highlighted in Fig. 5 for WWTP OT and WWTP AV. So overall, these micropollutants have in common that they are found at higher than average concentrations at WWTP AV, WWTP OT and WWTP SO.

These findings could be explained by the higher average age of the Northern Funen municipality compared to the other areas (Danmmarks Statistik 2021). WWTP SO and WWTP OT are both situated in Northern Funen municipality, where the average age in 2020 was 44.6 years. WWTP EM, WWTP NV and WWTP NE were placed in Odense municipality with an average age of 39.7 years and WWTP LY, WWTP DA and WWTP AV were placed around the Copenhagen area with an average age of 36.1 years (Danmmarks Statistik 2021). WWTP AV covers a larger part of municipalities not located in Copenhagen, and opposite to WWTP LY and WWTP DA. Where the average age in Copenhagen is 36.1 years, the average age in the towns discharging wastewater to WWTP AV (Albertslund, Ballerup, Brøndby, Glostrup, Høje-Taastrup, Ishøj and Vallensbæk) is between 39.4 and 42.2 years (Danmmarks Statistik 2021).

Nothern Funen municipality is split into multiple Parish districts. For Søndersø Parish district 30% of the population is above 60 years. For the Otterup Parish district, 34% of the population is above 60 years (Danmmarks Statistik 2021). The water inflow for WWTP OT was 2714 m^3^ day^−1^ and 1453 m^3^ day^−1^, for samples OT0714 and OT0721, respectively. For WWTP SO, the water inflow was 2714 m^3^ day^−1^ and 2126 m^3^ day^−1^, for SO0714 and SO0721, respectively (see Table S3). With fewer people in the catchment area, 9163 persons for WWTP SO compared to 10,112 persons for WWTP OT, this could suggest that WWTP SO receives a more significant portion of wastewater from sources such as industry and landfills. This could explain the observed differences between WWTP OT and WWTP SO that are located in comparable areas.

Irbesartan is also used for the treatment of high blood pressure. However, it is usually used in the initial treatment of high blood pressure (ASHP 2020). Irbesartan was measured at higher concentrations at WWTP AV than the other WWTPs. However, it was not detected at WWTP OT as the other blood pressure pharmaceuticals. One explanation for this could be differences in prescription. WWTP AV is in the capital region and WWTP OT is in the region of southern Denmark. Based on the sale numbers of the different regions (Table S6); furosemide, losartan and metoprolol are all prescribed 1.6, 1.4 and 1.2 times more often in the region of southern Denmark compared to the capital region. However, irbesartan is prescribed 0.6 times in the region of southern Denmark compared to the capital region, which could explain the observation.

Amitriptyline and citalopram, which are both pharmaceuticals used to treat depression, were found to have a Pearson correlation coefficient of 0.87 in effluent wastewater (SI_B, “Pearson Correlation”). For WWTP DA, average measured concentrations of amitriptyline and citalopram in effluent wastewater were 0.39 µg L^−1^ (range: 0.31 to 0.45 µg L^−1^) and 0.51 µg L^−1^ (range: 0.23 to 0.77 µg L^−1^), respectively (highlighted in Fig. 5). The average concentration of amitriptyline was 0.09 µg L^−1^ across all WWTPs and the range excluding WWTP DA was between 0.01 and 0.09 µg L^−1^. For citalopram the average concentration across all WWTPs was 0.16 µg L^−1^; excluding WWTP DA the concentration was between 0.05 and 0.17 µg L^−1^. The same clear trend was not observed for influent wastewater (Fig. S6). For amitriptyline, there were observed differences at WWTP DA in the concentrations in the effluent compared to influent wastewater compared to the other WWTPs as explained in “Fate of micropollutants in WWTPs.” It has not been possible to find an explanation for these observations of citalopram and amitriptyline.

Venlafaxine correlated with mirtazapine, sulfapyridine, propranolol and carbamazepine with a Pearson correlation coefficient of 0.90, 0.83, 0.85 and 0.82, respectively. Mirtazapine and venlafaxine are both anti-depressive pharmaceuticals; sulfapyridine is an antibiotic; propranolol is a beta blocker used to treat various illnesses; and carbamazepine has different use but is used to treat bipolar disorder (ASHP 2020). Venlafaxine correlates with O-desmethylvenlafaxine with a Pearson correlation coefficient of 0.95, which was expected since O-desmethylvenlafaxine is most likely a transformation product of venlafaxine (Gasser et al. 2012). These micropollutants were quantified at the highest concentrations at WWTP NE and WWTP OT (highlighted in Fig. 5). Venlafaxine, mirtazapine, propranolol and carbamazepine, for which sale numbers are available, have in common that they are prescribed 1.6 times more often in the region of southern Denmark compared to the capital region (Sundhedsdatastyrelsen 2021.). Both WWTP NE and WWTP OT are placed in the region of southern Denmark.

1,3-Diphenylguanidine is a vulcanisation agent that has been found to be leaching from tires (Zahn et al. 2019). 1,3-Dipehnylguanidine was detected in all WWTPs (Fig. 2). Before sampling, there were some rain events in Copenhagen (DMI 2022) with 2.8 mm on June 18, 12.1 mm on June 19 and 26.1 mm on June 20. Rain was measured again on June 28 with 4.1 mm, June 30 with 8.4 mm and July 1 with 2.3 mm. The samples taken on June 22, June 29 and July 1 from the Copenhagen-based WWTPs showed the highest concentration of 1,3-diphenylguanidine in effluent wastewater. The same trend is observed in the influent wastewater, with highest concentrations of the three Copenhagen-based WWTPs measured on 0629, and 0701, which could indicate that 1,3-diphenylguanidine is washed off from roads during rain events and ends up in wastewater, where it previously has been reported (Johannessen and Metcalfe 2022).

The most abundant pesticides (propiconazole, terbutryn and tebuconazole) have in common that they have also been used as biocides (Bester, Vollertsen, and Bollmann 2014). Propiconazole and tebuconazole are used in wood preservatives and terbutryn is used in painting. These micropollutants have been shown to leach from houses’ exterior surfaces during rain events (Bester, Vollertsen, and Bollmann 2014).

A Pearson correlation of 0.84 was calculated between propiconazole and 1,3-diphenylguanide. Similar, 1,3-diphenylguanide and terbutryn were found to have a Pearson correlation of 0.72. This corresponds well with the hypothesis of these micropollutants being washed off during rain events.

The correlation between tebuconazole was 0.08 and 0.07 for propiconazole and terbutryn, respectively. No micropollutants correlated with tebuconazole (Pearson correlation coefficient < 0.4). Tebuconazole is one of the fungicides used in the highest amounts with around 81,000 kg consumed in 2017, compared to 10,900 kg of propiconazole consumed in 2017 (SEGES 2021). Others found tebuconazole in a comparable amount in wastewater under dry and wet conditions (Bollmann et al. 2014). This suggests that the release of tebuconazole is not correlated to rain events.

Terbutryn was above the LOQ for all influent samples between 11 and 59 ng L^−1^, except for the influent sample taken on June 29 from WWTP DA, where a concentration of 509 ng L^−1^ was measured (Fig. S6). The remaining four influent samples from WWTP DA show concentrations of terbutryn in the range of 14 to 59 ng L^−1^. The spike in concentration on June 29 is also shown to influence the effluent concentrations. For WWTP DA, the highest effluent concentrations were measured on June 29 and July 1, as 106 and 156 ng L^−1^, respectively, compared to a concentration of 36 and 41 ng L^−1^, on July 22 and July 24, respectively. With a calculated hydraulic retention time of 46 h (Table S2), this corresponds with the spike’s influence on the effluent wastewater on July 1. This further highlights the issues of comparison of water packages of influent and effluent wastewater. The increased effluent concentrations measured in effluent wastewater on June 29 and July 1 suggest that the spike in influent concentration on June 29 is distributed out on different days in the effluent wastewater. This supports the findings from the lithium spike experiments made by Ejhed et al. (2018), which were discussed in “Fate of micropollutants in WWTPs.”

Furthermore, the concentration spike of terbutryn on June 29 shows how events can significantly change the risk assessment of the wastewater. Terbutryn was found to have the lowest PNEC of 2 ng L^−1^ for *R. subcapitata*. Routine measurements can easily miss such spikes of almost a factor 10 in concentration.

### Influent wastewater massflow estimated based on sale numbers and excretion

**Fig. 6:**
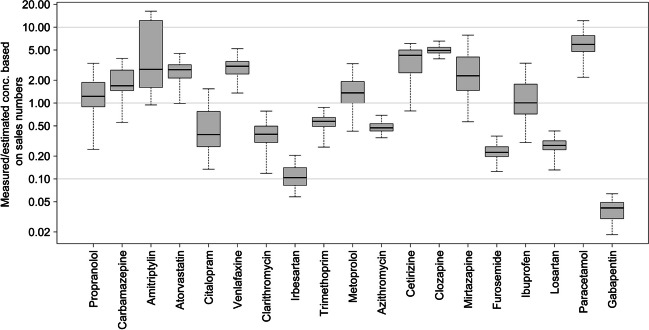
The amount in wastewater divided by the theoretical amount. The amount in wastewater is based on Eq. (S2), and the theoretical amount is based on sale numbers multiplied by the theoretical excretion. The boxplot is constructed based on the measurements in WWTPs in the Copenhagen and Odense area and the range of expected excretion. The pharmaceuticals are plotted from the lowest theoretical concentrations (left) to the highest expected concentrations (right)

Sale numbers of pharmaceuticals were combined with excretion to calculate an estimated concentration in influent wastewater. The measured influent wastewater concentration was divided with this estimated wastewater concentration and is presented in Fig. 6. The median influent concentration divided by theoretical concentration is within an order of 10 for 18 out of 19 investigated pharmaceuticals.

Among the compared theoretical and measured amounts, only gabapentin exhibited a deviation of more than an order of magnitude, with median concentrations at 0.04 times the expected concentration. The gabapentin recovery of the ml-SPE extraction method was found to be 14% (Table S6).

Irbesartan has a median influent concentration of 0.1 times the expected concentration based on sale numbers. Approximately 80% of irbesartan is excreted in feces (Wishart et al. 2018). Irbesartan has a log kow of 5.31 (KOWWIN v1.67 estimate), therefore it can be speculated that irbesartan is adsorbed in the wastewater sludge.

Some of the variations observed could potentially be explained by seasonal variation. The sale data is an average of yearly data, that would hide seasonal fluctuation, whereas the wastewater samples were collected during the summer of 2020. This could be the case for clarithromycin and azithromycin, and trimethoprim are all used as antibiotics and had a median of 0.39, 0.47 and 0.57 times the expected concentration, respectively. It is known that more antibiotic medicine is prescribed during winter compared to summer (Suda et al. 2014). Oppositely, cetirizine was found with a median of 4.2 times higher concentrations than expected. Cetirizine is an antihistamine for which it could be expected that there are seasonal fluctuations in use, with higher consumption in the spring/summer period (Medicin.dk 2019).

Disposal of pharmaceuticals to the toilet has previously been shown for antipsychotic pharmaceuticals such as carbamazepine (Ruhoy and Daughton 2008). With a low excretion of 3%, one dose directly disposed to the toilet will significantly influence the influent mass flow.

The model is based on the excretion numbers being so close as possible to the average excretion values. However, as previously also discussed by Escolà Casas et al. (2021), the access to excretion data is limited and in some cases excretion studies are made on a small number of people. Mirtazapine is found to have a median influent concentration that is 2.3 times higher than expected. For mirtazapine, a low excretion of 1–4% unchanged mirtazapine (Delbressine et al. 1998) has been found in literature and used for the calculations. However, the excretion value used is based on a study that investigated the excretion of four males and two females.

For paracetamol, the median influent concentration is 5.9 times higher than the expected value, which has been previously observed in literature (Escolà Casas et al. 2021). However, these studies have been based on prescription data and therefore expect such observation due to the high over-the-counter sale. The use of an excretion rate of 2–5% unchanged paracetamol may contribute to the overestimation (Escolà Casas et al. 2021).

Degradation through the sewage system can result in lower values in the wastewater. Jelic et al. (2015) have shown that the concentration of citalopram and clarithromycin decreases between 25 and 65% through a pressurised sewer with a retention time of 21 ± 2 h (Jelic et al. 2015).

Some pharmaceuticals are sold in larger quantities than needed for most common treatments. It is known that this is often the case for blood regulation medicine (Oosterhuis et al. 2013). This could explain the lower values found for furosemide and losartan, which fits with the results of previous studies that also found the wastewater concentrations to be below expected for these micropollutants (Oosterhuis et al. 2013).

Although there are limitations to this approach, with 18 out of 19 investigated pharmaceuticals within expected values, the use of excretion and sale data combined with PNEC can serve as a useful initial risk assessment. However, improving the quality of excretion studies is necessary to enhance the accuracy of pharmaceutical concentration estimations in wastewater.

## Conclusions

This study has shown the strength of comprehensive target analysis of micropollutants in wastewater from different WWTPs. We were able to show the presence of 79 different micropollutants in influent and effluent wastewater.

The main conclusions of this study:

- No significant differences were observed in the differences of influent and effluent wastewater concentrations of micropollutants among the investigated WWTPs. The similarity in WWTP design suggests that the removal from wastewater of specific micropollutants is primarily determined by their inherent properties rather than the specific characteristics of the WWTP.
- This study identified a potential risk associated with the release of wastewater to *D. Magna* and *R. subcapitata*. However, the calculation of predicted no-effect concentration (PNEC) was found to heavily influence the conclusions of the risk assessment. Further work is needed to establish standardized methodologies for calculating risk quotient (*RQ*) and conducting environmental risk assessments to ensure consistent results across studies.
- The composition of micropollutants in wastewater was found to be influenced by the catchment area, specifically the population residing in the area. Areas with an older population exhibited higher concentrations of age-specific medicines. Additionally, certain micropollutants were only detected in specific WWTPs e.g. azoxystrobin, metribuzin, metconazole and paclobutrazole indicating specific sources.
- The study highlights the potential of using wastewater for wastewater-based epidemiology, as the concentration of pharmaceuticals in influent wastewater could be predicted based on sale and excretion data. However, limited data availability on pharmaceutical excretion poses a limitation to these wastewater-based models. Improved data on pharmaceutical excretion is necessary to enhance the accuracy and reliability of such models in the future.

### Supplementary information

Below is the link to the electronic supplementary material.

Supplementary file1 (DOCX 280 KB)

Supplementary file2 (XLSM 255 KB)

## Data Availability

All the raw data are available and can be provided at reasonable request to the corresponding author.
