# Supplementary material for: Catchment area, fate, and environmental risks investigation of micropollutants in Danish wastewater
Source: Environ Sci Pollut Res Int. 2023 Nov 10;30(57):121107–23. doi: 10.1007/s11356-023-30331-z (PMC10698095; doi:10.1007/s11356-023-30331-z)
Supplement: Supplementary file 1 — Supplementary file1 (DOCX 280 KB) [file 11356_2023_30331_MOESM1_ESM.docx]

Supplementary: Catchment area, fate, and environmental risks investigation of micropollutants in wastewater

Kristoffer Kilpinen^1,2*^, Jason Devers^1^, Mafalda Castro^3^, Selina Tisler^1^, Mathias B. Jørgensen^4^, Peter Mortensen^2^, Jan H. Christensen^1^

1. Analytical Chemistry group, Department of Plant and Environmental Sciences, University of Copenhagen, Thorvaldsensvej 40, DK-1871 Frederiksberg C, Denmark
2. Eurofins Environment Denmark, Ladelundvej 85, DK-6600 Vejen, Denmark
3. Environmental Toxicology, Department of Plant and Environmental Science, University of Copenhagen, Thorvaldsensvej 40, DK-1871 Frederiksberg C, Denmark
4. BIOFOS a/s Refshalevej 250, DK-1432 Copenhagen, Denmark

Corresponding Author

* Corresponding author: kristofferkilpinen@eurofins.com, +45 26 86 42 11

Contents

[Supplementary materials for: Catchment area investigation, removal efficiency, and environmental risks of micropollutants in wastewater from a wide selection of wastewater treatment plants. 1](#_Toc115097155)

[1 Experimental 2](#_Toc115097156)

[1.1 Wastewater treatment plants (WWTPs) 2](#_Toc115097157)

[1.1.1 WWTP Lynetten (WWTP LY) 3](#_Toc115097158)

[1.1.2 WWTP Damhusåen (WWTP DA): 3](#_Toc115097159)

[1.1.3 WWTP Avedøre (AV): 3](#_Toc115097160)

[1.1.4 WWTP Ejby Mølle (EM), WWTP Nordvest (NV), and WWTP Nordøst (NE) 4](#_Toc115097161)

[1.1.5 WWTP Søndersø (SO) and WWTP Otterup (OT) 4](#_Toc115097162)

[1.2 Instrumental analysis and quantification 4](#_Toc115097163)

[1.2.1 LC-HRMS analysis 4](#_Toc115097164)

[1.2.2 GC×GC-QTOF analysis 5](#_Toc115097165)

[1.3 Sample specific metadata 5](#_Toc115097166)

[1.4 Sample preparation 7](#_Toc115097167)

[1.4.1 Internal standard and relative enrichment 7](#_Toc115097168)

[1.5 Quality control 10](#_Toc115097169)

[1.5.1 LC-QTOF 10](#_Toc115097170)

[1.5.2 GC×GC-QTOF 10](#_Toc115097171)

[1.6 Calculation of pharmaceutical consumptions 10](#_Toc115097172)

[1.7 Pharmaceutical consumption and excretion 11](#_Toc115097173)

[2 Results 13](#_Toc115097174)

[2.1 QC 13](#_Toc115097175)

[2.2 Recovery experiment SPE 14](#_Toc115097176)

[2.3 Investigation of recoveries 16](#_Toc115097177)

[2.4 Investigation of catchment areas 16](#_Toc115097178)

# Experimental

## Wastewater treatment plants (WWTPs)


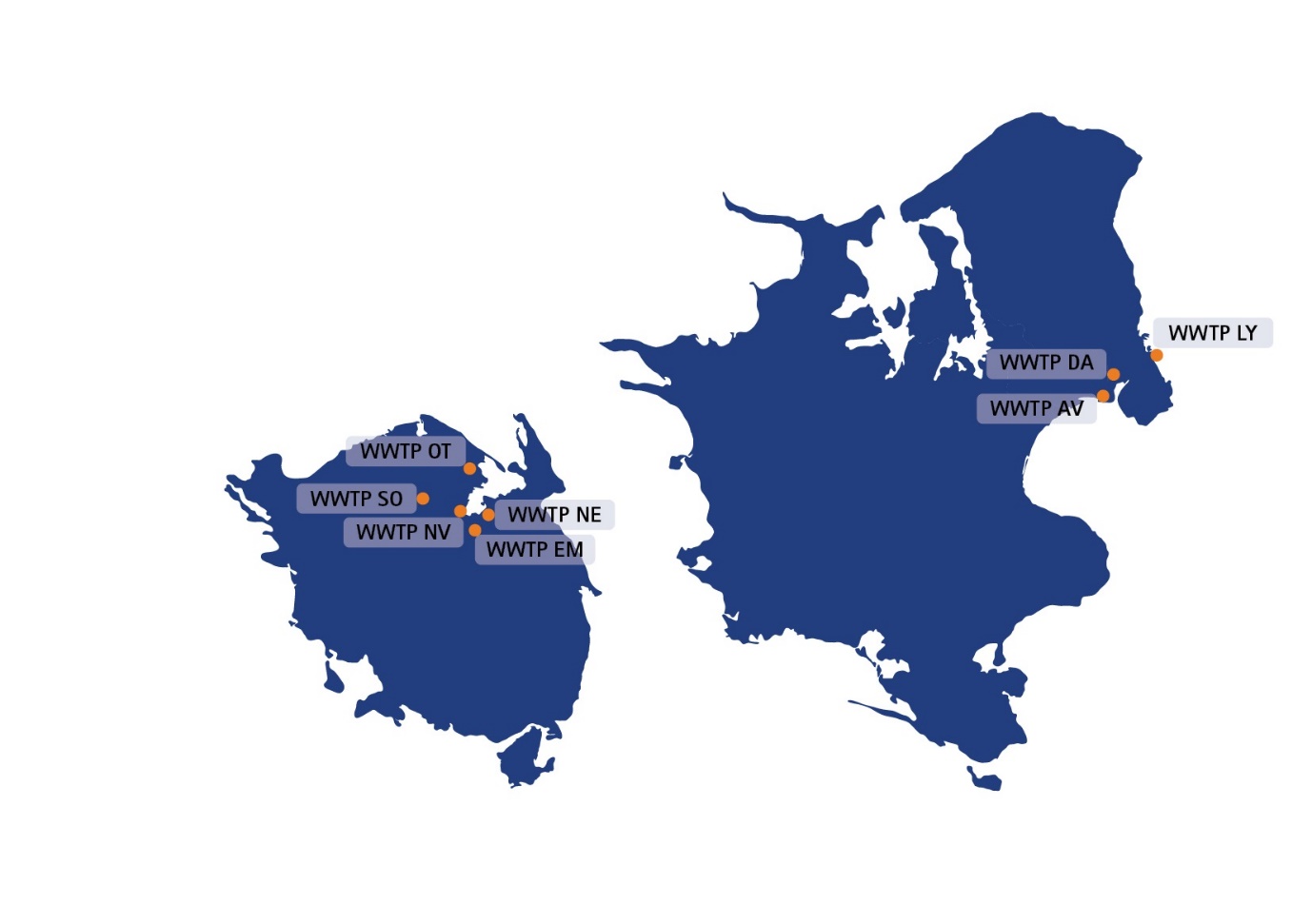


Fig. S1: Map of the WWTPs included in this study.

All wastewater treatment plants (WWTPs) are placed in Denmark, as according to Fig. S1. The WWTPs used similar techniques for cleaning wastewater. All of the WWTPs uses an initial mechanical filtration (M), followed by biological treatment (B), oxidation of ammonium to nitrate (N), and a chemical treatment (K) process. Some of the treatment plants then have an additional filtration step through a sand filter (F), while some of the treatment plants uses a large water reservoir (L). See Table S1 for details on specific treatment steps used in each of the WWTPs.

Table S1: Overview of the samples included in this study. The WWTP, the location, treatment steps, the estimated capacity of the WWTP in PE, and the number of 24-hour composite influent and effluent samples collected for this study. Number of people connected to the WWTP are based on numbers from DST “Det danske kvadratnet” (GIS system developed of DST).

| Full name | WWTP | Location | Treatment steps in WWTP | Capacity (PE) | Number of influent samples | Number of effluent samples | Number of people |
| --- | --- | --- | --- | --- | --- | --- | --- |
| Rensningsanlæg Lynetten | WWTP LY | Urban/Copenhagen | MBNDK | 750,000 | 7 | 4 | 643,746 |
| Rensningsanlæg Avedøre | WWTP AV | Urban/Copenhagen | MBNDK | 400,000 | 2 | 4 | 249,395 |
| Rensningsanlæg Damhusåen | WWTP DA | Urban/Copenhagen | MBNDKF | 350,000 | 5 | 4 | 286,562 |
| Rensningsanlæg Ejby Mølle | WWTP EM | Urban/Odense | MBNDKF | 385,000 | 4 | 4 | 126,432 |
| Rensningsanlæg Nordvest | WWTP NV | Urban/Odense | MBNDKF | 48,300 | 0 | 2 | 49,752 |
| Rensningsanlæg Nordøst | WWTP NE | Urban/Odense | MBNDKF | 30,000 | 2 | 4 | 29,133 |
| Rensningsanlæg Søndersø | WWTP SO | Rural/Northern Fyn | MBNDKL | 20,000 | 0 | 2 | 9,163 |
| Rensningsanlæg Otterup | WWTP OT | Rural/Northern Fyn | MBNDKL | 12,500 | 0 | 2 | 10,112 |

A more detailed description of WWTPs included in this study is given below.

### WWTP Lynetten (WWTP LY)

WWTP LY is placed in the Copenhagen area. WWTP LY is a part of BIOFOS that treats wastewater from the municipals around the Copenhagen area. The municipals that contribute with wastewater to WWTP LY are Copenhagen, Frederiksberg, and Gentofte that has average ages of 36.1, 40.0, and 42.5 years, respectively (Danmmarks Statistik, 2021). In the catchment area lives an estimated 643,746 people.

Hospitals in the catchment area includes: Amager Hospital, Bispebjerg Hospital, Rigshospitalet, Frederiksberg Hospital, and Gentofte Hospital.

### WWTP Damhusåen (WWTP DA):

WWTP DA is placed in the Copenhagen area and operated by BIOFOS. The municipals that contribute with wastewater to the WWTP are Copenhagen and Frederiksberg. For Copenhagen municipal the average age are 36.1 years and for Frederiksberg Municipal the average age is 40.0 years (Danmmarks Statistik, 2021). In the catchment area lives an estimated 286,562 people.

Hvidovre Hospital is placed in the catchment area not including Hvidovre Hospitals psychiatric department.

### WWTP Avedøre (WWTP AV):

WWTP AV is placed in the Copenhagen area and operated by BIOFOS. The municipals that contribute with wastewater to the WWTP are Copenhagen with an average age of 36.1 years but there is also contribution from Albertslund, Ballerup, Brøndby, Glostrup, Høje-Taastrup, Ishøj and Vallensbæk where the average age is between 39.4 and 42.2 years (Danmmarks Statistik, 2021).

Of industry in the area can be mention a manufacture of vitamin and polyunsaturated fatty acids, a manufacture of candy and a pharmaceutical manufacture.

Herlev hospital is placed in the catchment area together with Hvidovre Hospitals psychiatric department.

### WWTP Ejby Mølle (WWTP EM), WWTP Nordvest (WWTP NV), and WWTP Nordøst (WWTP NE)

WWTP EM, WWTP NV, and WWTP NE are placed in Odense Municipal and operated by Vandcenter Syd. Odense municipal has an average age of 39.7 years (Danmmarks Statistik 2021).

For WWTP EM, Odense Universitets Hospital are placed in the catchment area. Of industries can be mention: a brewery, a marzipan factory, detergent factory, a washer, and greenhouses. In the catchment area lives an estimated 126,432 people.

For WWTP NV, industries include: landfill, metal processing factory and greenhouses. In the catchment area lives an estimated 49,752 people.

For WWTP NE, industries include: recipient of contaminated soil and waste, datacenter, cheese dairy and greenhouses. In the catchment area lives an estimated 29,133 people.

### WWTP Søndersø (WWTP SO) and WWTP Otterup (WWTP OT)

WWTP SO and WWTP OT are placed in Nordfyns municipal and is operated by Vandcenter syd. Nordfyns municipal has an average age of 44.6 years (Danmmarks Statistik, 2021).

For WWTP SO, industries include: chips and peanuts manufactory, feed manufactory, foam plastic and polyurethane manufactory, and greenhouses. In the catchment area lives an estimated 9,163 people.

For WWTP OT, industries include: Manufacture of rapeseed oil, rapeseed oil methyl ester, glycerin and feed cakes from rapeseed, recipient of crops, and greenhouses. In the catchment area lives an estimated 10,112 people.

## Instrumental analysis and quantification

### LC-HRMS analysis

The samples were analysed on an Acquity Ultra-Performance Liquid Chromatograph (UPLC) equipped with a Synapt G2S quadrupole time of flight mass spectrometry (QTOFMS) (Waters, Taastrup, Denmark). The samples were injected in both positive and negative electrospray ionisation mode. The instrument was equipped with a 2.1 inner diameter x 100 mm length Acquity UPLC BEH C18 column with 1.7 µm particles (Waters, Taastrup, Denmark). 2 µl of the diluted ml-SPE extract sample (REF 10 or REF 50) was injected and analysed using a flow of 0.3 ml min^-1^ with mobile phase A: 0.1 % formic acid in water and mobile phase B: 0.1 % formic acid in acetonitrile. A gradient was used, with 99 % A and 1 % B kept for the first minute, then going to 70 % A and 30 % B for 3 minutes, then going with a gradient to 1 % A and 99 % B at 16 minutes, these settings were kept for 5 minutes (21 minutes), at which the system was changed to 99 % A and 1 % B and conditioned for 4 minutes. The data was obtained using MS full scan and MS^e^ (data independent fragmentation). With a scan time of 0.35 s, and a ramp trap collision energy of 10 to 40 eV.

During the analysis, lock spray calibration was performed using leucine-enkephalin. Lock mass 556.2771 Da and mass 554.2615 Da were used for positive and negative ionization, respectively. The LC-HRMS extracted ion chromatograms (EICs) were extracted with ±0.1 da and inspected in TargetLynx (Waters, Taastrup, Denmark) and UNIFI (Waters, Taastrup, Denmark) for comparison of fragmentation patterns. The peak areas were exported to Excel (Microsoft, Redmond, USA), where the data was quantified using linear internal standard calibration.

### GC×GC-QTOF analysis

Samples were analysed on an Agilent 7890B GC system equipped with an Agilent 7200 Accurate Mass QTOFMS (Agilent Technology, Palo Alto, CA, USA). Two-dimensional separation was carried out using a secondary column oven paired with a Zoex ZX2 cryogen-free modulator (Zoex Corporation, Houston, TX, USA). A 60 m, 0.25 mm id, 0.25 µm non-polar ZB-5 column (Phenomenex, Torrance, CA, USA) was used in first dimension, and a 1.5 m, 0.18 mm i.d., 0.18 µm mid-polar ZB-50 column (Phenomenex, Torrance, CA, USA) was used for the second dimension.

For effluent samples, 5 µl of samples with REF 450 were injected using a solvent vent method with an initial inlet temperature of 60 ^o^C, ramped to 280 ^o^C at a rate of 600 ^o^C/min after 1.1 min. For influent samples, 1 µl of the sample was injected in splitless mode with an inlet temperature of 280 ^o^C. For both influent and effluent samples, standards were injected under similar conditions.

The primary oven temperature followed the program: 60 °C for 1 minute, ramped to 315 °C at a rate of 3 °C/min, with a final hold of ten minutes (total run time: 96 minutes). The secondary oven and hot jet temperature were operated at a constant temperature offset of +60 °C to the primary oven, with the column temperature plateauing at 315 °C and the hot jet at 360 °C. Helium was used as the carrier gas with a constant flow rate of 2.0 mL/min. The modulation period was set to 7000 ms, with a hot jet on-time of 750 ms. The chromatograms were extracted with ±0.05 da and analysed using GC image (GC Image LLC, version 2.9R1.1). A template was generated, including template objects for each analyte, including quantification ions, qualification ions, and retention times.

The samples were quantified using internal standard calibration. For each analyte, a 2^nd^-degree polynomial calibration curve was made in GC image and used for the quantification.

## Sample specific metadata

The retention time of wastewater in the WWTP were calculated based on the Inlet water flow and the size of the reservoirs. The total retention time of the wastewater was estimated based on equation S1.

Equation S1

$$\boldsymbol{Wastewater Retention Time}\mathbf{=}\frac{\mathbf{Reservoir Volume}}{\mathbf{Flow}}\boldsymbol{\cdot}\mathbf{24 h}$$

The Wastewater Retention Time was calculated for various steps in the WWTP. It was calculated by dividing the Reservoir Volume (m^3^) with the Flow (m^3^/day) multiplied with 24 hours. For water flows and wastewater retention calculation, see table S2.

Table S2: Overview of the samples analyzed in this study. For WWTP LY, WWTP OT and WWTP SO, outflow water flow were not available (n.a.).

| Sample ID | Type | Date (mmdd-2020) | WWTP | Sampling date (Start date) | Sampling time | Inlet water flow (m^3^ day^-1^) | Outlet water flow (m^3^ day^-1^) | Bypass (m^3^ day^-1^) | Retention time in bio (hours) | Total retention time (hours) |
| --- | --- | --- | --- | --- | --- | --- | --- | --- | --- | --- |
| LY0622 | Influent | 0622 | LY | 2020-06-22 | 24 hr | 137126 | n.a. | 0 | 11 | 40 |
| LY0622 | Influent | 0622 | LY | 2020-06-22 | 24 hr | 137126 | n.a. | 0 | 11 | 40 |
| AV0622 | Influent | 0622 | AV | 2020-06-22 | 24 hr | 49783 | 54253 | 0 | 20 | 67 |
| DA0622 | Influent | 0622 | DA | 2020-06-22 | 24 hr | 67282 | 67134 | 0 | 10 | 39 |
| DA0622 | Influent | 0622 | DA | 2020-06-22 | 24 hr | 67282 | 67134 | 0 | 10 | 39 |
| LY0622 | Effluent | 0622 | LY | 2020-06-22 | 24 hr | 137126 | n.a. | 0 | 11 | 40 |
| AV0622 | Effluent | 0622 | AV | 2020-06-22 | 24 hr | 49783 | 54253 | 0 | 20 | 67 |
| DA0622 | Effluent | 0622 | DA | 2020-06-22 | 24 hr | 67282 | 67134 | 0 | 10 | 39 |
| LY0624 | Influent | 0624 | LY | 2020-06-24 | 24 hr | 125728 | n.a. | 0 | 12 | 43 |
| LY0624 | Influent | 0624 | LY | 2020-06-24 | 24 hr | 125728 | n.a. | 0 | 12 | 43 |
| DA0624 | Influent | 0624 | DA | 2020-06-24 | 24 hr | 51800 | 51611 | 0 | 12 | 51 |
| LY0624 | Effluent | 0624 | LY | 2020-06-24 | 24 hr | 125728 | n.a. | 0 | 12 | 43 |
| AV0624 | Effluent | 0624 | AV | 2020-06-24 | 24 hr | 46858 | 48333 | 0 | 22 | 71 |
| DA0624 | Effluent | 0624 | DA | 2020-06-24 | 24 hr | 51800 | 51611 | 0 | 12 | 51 |
| LY0629 | Influent | 0629 | LY | 2020-06-29 | 24 hr | 143534 | n.a. | 0 | 10 | 38 |
| LY0629 | Influent | 0629 | LY | 2020-06-29 | 24 hr | 143534 | n.a. | 0 | 10 | 38 |
| DA0629 | Influent | 0629 | DA | 2020-06-29 | 24 hr | 56856 | 56115 | 0 | 11 | 46 |
| LY0629 | Effluent | 0629 | LY | 2020-06-29 | 24 hr | 143534 | n.a. | 0 | 10 | 38 |
| AV0629 | Effluent | 0629 | AV | 2020-06-29 | 23 hr 50 min | 49738 | 42370 | 0 | 20 | 67 |
| DA0629 | Effluent | 0629 | DA | 2020-06-29 | 24 hr | 56856 | 56115 | 0 | 11 | 46 |
| LY0701 | Influent | 0701 | LY | 2020-07-01 | 24 hr | 194522 | n.a. | 63 | 7 | 28 |
| AV0701 | Influent | 0701 | AV | 2020-07-01 | 24 hr | 65142 | 64280 | 0 | 15 | 51 |
| DA0701 | Influent | 0701 | DA | 2020-07-01 | 24 hr | 98228 | 95306 | 2375 | 7 | 27 |
| LY0701 | Effluent | 0701 | LY | 2020-07-01 | 24 hr | 194522 | n.a. | 63 | 7 | 28 |
| AV0701 | Effluent | 0701 | AV | 2020-07-01 | 24 hr | 65142 | 64280 | 0 | 15 | 51 |
| DA0701 | Effluent | 0701 | DA | 2020-07-01 | 24 hr | 98228 | 95306 | 2375 | 7 | 27 |
| EM0707 | Effluent | 0707 | EM | 2020-07-07 | 24 hr | 37100 | 34300 | 0 | 33 | 38 |
| NV0707 | Effluent | 0707 | NV | 2020-07-07 | 24 hr | 16644 | 15846 | 0 | 20 | 35 |
| NE0707 | Effluent | 0707 | NE | 2020-07-07 | 24 hr | 4484 | 4484 | 0 | 22 | 41 |
| EM0714 | Influent | 0714 | EM | 2020-07-14 | 24 hr | 42500 | 39800 | 0 | 29 | 33 |
| EM0714 | Influent | 0714 | EM | 2020-07-14 | 24 hr | 42500 | 39800 | 0 | 29 | 33 |
| NE0714 | Influent | 0714 | NE | 2020-07-14 | 24 hr | 4454 | 4454 | 0 | 23 | 41 |
| EM0714 | Effluent | 0714 | EM | 2020-07-14 | 24 hr | 42500 | 39800 | 0 | 29 | 33 |
| NE0714 | Effluent | 0714 | NE | 2020-07-14 | 24 hr | 4454 | 4454 | 0 | 23 | 41 |
| OT0714 | Effluent | 0714 | OT | 2020-07-14 | 18 hr | 2714 | n.a. | n.a. | n.a. | n.a. |
| SO0714 | Effluent | 0714 | SO | 2020-07-14 | 24 hr | 2176 | n.a. | n.a. | n.a. | n.a. |
| EM0716 | Influent | 0716 | EM | 2020-07-16 | 24 hr | 28800 | 27300 | 0 | 42 | 49 |
| EM0716 | Influent | 0716 | EM | 2020-07-16 | 24 hr | 28800 | 27300 | 0 | 42 | 49 |
| NE0716 | Influent | 0716 | NE | 2020-07-16 | 24 hr | 3623 | 3623 | 0 | 28 | 51 |
| EM0716 | Effluent | 0716 | EM | 2020-07-16 | 24 hr | 28800 | 27300 | 0 | 42 | 49 |
| NV0716 | Effluent | 0716 | NV | 2020-07-16 | 24 hr | 8442 | 7972 | 0 | 40 | 70 |
| NE0716 | Effluent | 0716 | NE | 2020-07-16 | 24 hr | 3623 | 3623 | 0 | 28 | 51 |
| OT0721 | Effluent | 0721 | OT | 2020-07-21 | 24 hr | 1453 | n.a. | n.a. | n.a. | n.a. |
| EM0721 | Effluent | 0721 | EM | 2020-07-21 | 24 hr | 27100 | 25600 | 0 | 45 | 52 |
| NE0721 | Effluent | 0721 | NE | 2020-07-21 | 24 hr | 3545 | 3545 | 0 | 28 | 52 |
| SO0721 | Effluent | 0721 | SO | 2020-07-21 | 24 hr | 2126 | n.a. | n.a. | n.a. | n.a. |

## Sample preparation

For the sample preparation an automated SPE-03 system, PromoChrom Technologies Ltd (Richmond, Canada) was used. The Solid-phase- extraction (SPE) cartridges were prepared with 200 mg Supelclean ENVI-Carb from (Merck Darmstadt, Germany) in the bottom, followed by a layer consisting of 560 mg mixture of three sorbents in a ratio of 1:1:0.8. The three sorbents being Oasis Weak Anion Exchange (Waters, Tåstrup, Denmark), Cationic Exchange (Waters, Tåstrup, Denmark) and Isolute ENV+ (Biotage Uppsala, Sweden), respectively.

The cartridges were prepared with 5 ml of methanol, followed by 10 ml of water. The sample (1 L) was loaded onto the column at a rate of 6 mL min^-1^. After the sample was loaded to the column, the column was allowed to dry for 50 min. The column was then reverted and eluted from the bottom to the top. The column was eluted with first 6 ml ethyl acetate/methanol/25% ammonium hydroxide (46/46/8), secondly 3 mL ethyl acetate/methanol/formic acid (49/49/1.7) and finally 2 mL of methanol. The collected sample was evaporated at 40^o^C under a gentle nitrogen flow to approximately 300 µL, and reconstituted to 2 ml with methanol (Tisler et al., 2021).

### Internal standard and relative enrichment

The ml-SPE extracts and standards were spiked with internal standards. The reason for adding the internal standard at this stage and not before ml-SPE was that the extracts were divided after enrichment. One part was used for in-vitro and in-vivo effect studies (paper in preparation). Thus, internal standards could not be added to the samples before enrichment. Each internal standard was assigned to a micropollutant based on closed retention time (RT) (Table S3).

For LC-HRMS analysis, effluent samples were injected at REF 50, whereas influent samples were injected at REF 10. These REFs were selected as a compromise to reduce matrix effects without losing too much sensitivity and based on previous work from this group (Tisler et al., 2021). The samples were spiked to 4.5 µg L^-1^ of each internal standard for LC-HRMS analysis.

For GC×GC-QTOF analysis, the samples were spiked to 100 to 944 µg L^-1^ of each internal standard (Table S4) and analysed at REF 450.

**Table S3**: Internal standards (ISTD) used for correction of micropollutants (Analyte).

| Analyte | CAS-nr | Log Kow | ISTD |
| --- | --- | --- | --- |
| Mirtazapine | 61337-67-5 | 1.5 | Hydroxy-Atrazin-D5 |
| Irbesartan | 138402-11-6 | 3.9 | Simazin-D10 |
| 1,2,3-Benzotriazole | 95-14-7 | 1.4 | Hydroxy-Atrazin-D5 |
| Clozapine | 5786-21-0 | 3.2 | Hydroxy-Atrazin-D5 |
| Thiabendazole | 148-79-8 | 2.0 | Hydroxy-Atrazin-D5 |
| Boscalid | 188425-85-6 | 4.0 | Metolachlor-d6(propyl-d6) |
| Paclobutrazole | 266-325-7 | 3.4 | Terbuthylazine-d5(ethyl-d5) |
| Diatrizoic acid | 117-96-4 | 2.6 | Hydroxy-Atrazin-D5 |
| 1,3-Diphenylguanidine | 102-06-7 | 3.0 | Hydroxy-Atrazin-D5 |
| Losartan | 114798-26-4 | 4.0 | Atrazin-D5 |
| Isoproturon | 34123-59-6 | 2.7 | Isoproturon-D6 |
| N,N-Diethyl-3-methylbenzamide (DEET) | 134-62-3 | 2.2 | Isoproturon-D6 |
| Clarithromycin | 81103-11-9 | 3.2 | Simazin-D10 |
| Cetirizine | 83881-51-0 | 1.7 | Atrazin-D5 |
| Carbamazepine | 298-46-4 | 2.4 | Carbamazepine-D8 |
| Trimethoprim | 738-70-5 | 0.7 | Hydroxy-Atrazin-D5 |
| Epoxiconazole | 135319-73-2 | 3.5 | Metolachlor-d6(propyl-d6) |
| 4-(trifluoromethyl)-phenol (TFMP) | 33252-63-0 | 0.7 | Hydroxy-Atrazin-D5 |
| Acridine | 260-94-6 | 3.4 | Hydroxy-Atrazin-D5 |
| Atrazine | 1912-24-9 | 2.6 | Atrazin-D5 |
| Metconazole | 125116-23-6 | 4.2 | Metolachlor-d6(propyl-d6) |
| Metribuzin | 21087-64-9 | 1.5 | Simazin-D10 |
| Pirimicarb | 23103-98-2 | 1.4 | Hydroxy-Atrazin-D5 |
| Tebuconazol | 107534-96-3 | 3.7 | Metolachlor-d6(propyl-d6) |
| Sulfamethizol | 144-82-1 | 0.4 | Hydroxy-Atrazin-D5 |
| Propranolol | 318-98-9 | 0.2 | Simazin-D10 |
| Naproxen | 22204-53-1 | 3.2 | Terbuthylazine-d5(ethyl-d5) |
| Metoprolol | 37350-58-6 | 1.7 | Hydroxy-Atrazin-D5 |
| Diclofenac | 15307-86-5 | 4.5 | Metolachlor-d6(propyl-d6) |
| Erythrocin | 114-07-8 | 3.1 | Simazin-D10 |
| Azithromycin | 83905-01-5 | 4.0 | Hydroxy-Atrazin-D5 |
| Clindamycin | 18323-44-9 | 2.2 | Hydroxy-Atrazin-D5 |
| O-desmethylvenlafaxine | 93413-62-8 | 2.7 | Hydroxy-Atrazin-D5 |
| Citalopram | 59729-33-8 | 3.7 | Simazin-D10 |
| Valsartan | 137862-53-4 | 0.9 | Terbuthylazine-d5(ethyl-d5) |
| Amitriptyline | 50-48-6 | 5.0 | Simazin-D10 |
| Caffeine | 58-08-2 | 0.2 | Hydroxy-Atrazin-D5 |
| Azoxystrobin | 131860-33-8 | 1.6 | Metolachlor-d6(propyl-d6) |
| Gabapentin | 60142-96-3 | -1.1 | Hydroxy-Atrazin-D5 |
| Venlafaxine | 93413-69-5 | 3.1 | Venlafaxine-d6 |
| Terbutryn | 886-50-0 | 3.6 | Atrazin-D5 |
| Sulfapyridine | 144-83-2 | 0.4 | Hydroxy-Atrazin-D5 |
| Sulfamethoxazole | 723-46-6 | 0.9 | Simazin-D10 |
| Paracetamol | 103-90-2 | 0.5 | Hydroxy-Atrazin-D5 |
| MCPB | 94-81-5 | 3.3 | DNOC-D5 |
| Benzoic acid | 532-32-1 | -1.6 | Dichlorprop-D6 |
| Salicylic acid | 69-72-7 | 2.2 | Bentazon-D6 |
| MCPA | 94-74-6 | 2.5 | DNOC-D5 |
| Sucralose | 56038-13-2 | -0.7 | Hydroxy-Atrazin-D5 |
| Furosemide | 54-31-9 | 2.0 | Bentazon-D6 |
| Sulfadiazine | 68-35-9 | -0.1 | Dichlorprop-D6 |
| Atorvastatin | 134523-00-5 | 6.8 | Dichlorprop-D6 |
| Mecoprop | 93-65-2 | 3.1 | Dichlorprop-D6 |
| Saccharin | 81-07-2 | 0.5 | Hydroxy-Atrazin-D5 |
| 4-nitrophenol | 100-02-7 | 1.9 | Bentazon-D6 |
| Propiconazole | 60207-90-1 | 4.1 | Metolachlor-d6(propyl-d6) |
| Terbutylazin | 5915-41-3 | 3.2 | Hydroxy-Atrazin-D5 |
| Prosulfocarb | 52888-80-9 | 4.2 | Metolachlor-d6(propyl-d6) |
| Phenol | 108-95-2 | 1.5 | Phenol-D6 |
| Aniline | 2348-49-4 | 1.1 | Aniline-D5 |
| 3-Methylphenol | 108-39-4 | 1.9 | Phenol-D6 |
| 2-Phenoxyethanol | 122-99-6 | 1.1 | Phenol-D6 |
| Benzothiazole | 95-16-9 | 2.2 | Phenol-D6 |
| Benzeneacetic acid | 17303-65-0 | 1.4 | Phenol-D6 |
| Indole | 120-72-9 | 2.1 | Indole-D7 |
| Hydrocinnamic acid | 501-52-0 | 2.3 | Carbamazepine-D8 |
| 3-Methylindole | 83-34-1 | 2.6 | Indole-D7 |
| Butylated Hydroxytoluene | 128-37-0 | 5.0 | Phenol-D6 |
| Diethyl Phthalate | 84-66-2 | 2.7 | Phenol-D6 |
| Ibuprofen | 15687-27-1 | 3.8 | Ibuprofen-D3 |
| Tetradecanoic acid | 544-63-8 | 6.0 | Palmitic Acid-C13 |
| Pentachlorophenol | 87-86-5 | 4.7 | Ibuprofen-D3 |
| 4-nonyl phenol | 104-40-5 | 6.0 | Indole-D7 |
| Theobromine | 519-41-5 | -0.1 | Caffeine-D9 |
| 1,7-Dimethylxanthine | 611-59-6 | -0.4 | Caffeine-D9 |
| Lidocaine | 137-58-6 | 1.7 | Lidocaine-D10 |
| Dibutyl Phthalate | 84-74-2 | 4.6 | Fluoranthene-D10 |
| 9-Methylacridine | 611-64-3 | 3.9 | Lidocaine-D10 |
| Palmitic Acid | 57-10-3 | 7.0 | Palmitic Acid-C13 |
| Oleic Acid | 112-80-1 | 7.7 | Palmitic Acid-C13 |
| Fluoranthene | 206-44-0 | 4.9 | Fluoranthene-D10 |

Table S4: Concentrations of internal standards spiked to samples for GC-GC-QTOF analysis.

| Internal standard | Concentration in sample  (µg L^-1^) |
| --- | --- |
| Aniline-D5 | 598 |
| Phenol-D6 | 944 |
| Indole-D7 | 508 |
| Caffeine-D9 | 545 |
| Lidocaine-D10 | 575 |
| Fluoranthene-D10 | 100 |
| Carbamazepine-D8 | 250 |
| Ibuprofen-D3 | 580 |

## Quality assurance

The method was assessed with respect to the limit of quantification (LOQ), precision, linearity, method blanks and instrument blanks.

For LC-QTOF, the LOQ was estimated based on the external standards, and the LOQ was assigned as the lowest standard where the micropollutant had a signal-to-noise ratio above 10 (Hollender et al., 2018). The instrumental precision was investigated by injecting all samples three times. All results are reported as an average of these three measurements with an uncertainty based on these three measurements. For most micropollutants, the linearity was examined in the range of 0.05 µg L^-1^ to 100 µg L^-1^. For a complete list of standards and concentrations, see SI_B, “Standards”. Two method blanks were made using tap-water that was extracted and analysed together with the samples. Instrument blanks were made of methanol, spiked with internal standards and analysed together with the samples.

For GC×GC-QTOF, LOQ was determined based on the repeated preparation and injection of a low level standard (Level 3 in SI_B,”Standards”), for the 2nd-degree polynomial calibration curves, LOQ was determined as the concentration with a relative standard deviation equal to the required accuracy of 10%. QC samples were included for both LC-QTOF and GC×GC-QTOF and a detailed description has been given in S1.6

## Quality control

### LC-QTOF

For every four wastewater samples, a pooled QC sample was injected. The pooled QC sample used for effluent samples was prepared by combining 5 µL aliquot of all effluent samples. Similarly, the pooled QC sample used for influent samples was prepared by combining 5 µL aliquot of all influent samples. A combined QC sample (SuperQC) was prepared by mixing the influent and effluent QC sample (ration 1:4). The SuperQC was injected for every eight effluent or influent samples. These QC samples were analysed to monitor drifts within and between analytical batches.

### GC×GC-QTOF

For each four wastewater samples injected, a pooled QC sample was injected. Similar to the LC-QTOF experiment, the QC was comprised of pooled samples of either influent or effluent. QC samples were used to monitor signal drift within and between batches and to determine precision.

## Calculation of pharmaceutical consumptions

The theoretical concentration of pharmaceuticals in wastewater was calculated according to Equation S2 and the measured concentration was calculated according to Equation S3.

Equation S2:

$$Theoretical concentration=\frac{\mathrm{DDD} \left( mg {day}^{-1} \right)x Sold DDD x Excretion(\%)}{100 \% x 1000 persons {day}^{-1}}$$

here DDD is the amount in one daily dose, Sold DDD is the number of DDD sold for 1000 persons/day, excretion is the amount of active pharmaceutical excreted. The measured concentration in influent wastewater was calculated into a Measured concentration that could be used for comparison using equation 4.

Equation S3:

$$Measured concentration=\frac{Influent concentration \left( {mg L}^{-1} \right)x Flow(L {day}^{-1})}{Number of people connected to the WWTP}$$

Where Influent concentration is the measured concentration in wastewater, flow is the flow at the inlet of the WWTP (Table S2) and this is divided by the number of people connected to the WWTP (Table S1).

Data was transferred to Excel and a Monte Carlo Simulation was made using the high and low extraction. The simulation was made by using 100 guesses between high and low extraction for each included pharmaceuticals. The guessing was done using the built in Excel random function (0 to 1). Assuming the similar possibility of all outcomes within the model. This was combined with the measured concentrations of pharmaceuticals in a box plot model.

## Pharmaceutical consumption and excretion

Table S5: Pharmaceuticals used for the investigation of the comparison of theoretical versus measured concentration. For all pharmaceuticals the name of the pharmaceutical is given (Pharmaceutical), the cas-number of the pharmaceutical (CAS), the ATC code used for the calculation (ATC code), the amount of pharmaceutical per DDD according to WHOCC, DDD/1000 persons at Region Hovedstaden according to medstat, low excretion is the lowest excretion according to literature, high excretion is the highest excretion according to literature, reference are the reference used for excretion.

| Pharmaceutical | CAS | ATC Code | Amount of pharmaceutical per DDD (mg) | DDD/1000 persons Region Hovedstaden | DDD/1000 persons Region Syddanmark | Low Excretion (%) | High excretion (%) | Reference |
| --- | --- | --- | --- | --- | --- | --- | --- | --- |
| Amitriptyline | 549-18-8 | N06AA09 | 75 | 1.3 | 1.8 | 2 | 2 | (Wishart et al., 2018) |
| Atorvastatin | 134523-00-5 | C10AA05 | 20 | 94.4 | 120.2 | 2 | 2 | (Sonje et al., 2010) |
| Azithromycin | 83905-01-5 | J01FA10 | 300 | 0.4 | 0.4 | 53 | 59 | (Escolà Casas et al., 2021; Luke & Foulds, 1997; Wishart et al., 2018) |
| Carbamazepine | 298-46-4 | N03AF01 | 1000 | 0.5 | 0.9 | 3 | 3 | (Escolà Casas et al. 2021; FDA) |
| Cetirizine | 83881-51-0 | R06AE07 | 10 | 20.8 | 18.8 | 50 | 60 | (Aoshima et al., 2001; Portnoy & Dinakar, 2004) |
| Citalopram | 59729-33-8 | N06AB04 | 20 | 11.2 | 12.6 | 15 | 30 | (Dalgaard & Larsen, 1999; emc, 2022; Escolà Casas et al., 2021; Oyehaug et al., 1984; Wishart et al., 2018) |
| Clarithromycin | 81103-11-9 | J01FA09 | 500 | 0.4 | 0.3 | 22 | 40 | (Escolà Casas et al. 2021) |
| Clozapine | 5786-21-0 | N05AH02 | 300 | 0.6 | 0.7 | 2.5 | 2.5 | (Sheehan et al., 2010) |
| Furosemide | 54-31-9 | C03CA01 | 40 | 32.8 | 52.5 | 62 | 78 | (Escolà Casas et al. 2021) |
| Gabapentin | 60142-96-3 | N03AX12 | 1800 | 4 | 5.3 | 100 | 100 | (McLean 1994) |
| Ibuprofen | 15687-27-1 | M01AE01 | 1200 | 21.9 | 21.1 | 1 | 12 | (Escolà Casas et al. 2021) |
| Irbesartan | 138402-11-6 | C09CA04 | 150 | 0.7 | 0.4 | 74 | 76 | (Escolà Casas et al. 2021; Israili 2000a) |
| Losartan | 114798-26-4 | C09CA01 | 50 | 68.5 | 83.6 | 55 | 69 | (Escolà Casas et al. 2021; Israili 2000b) |
| Metoprolol | 56392-17-7 | C07AB02 | 150 | 18 | 26.4 | 3 | 11 | (Escolà Casas e  t al. 2021) |
| Mirtazapine | 61337-67-5 | N06AX11 | 30 | 5.7 | 9.8 | 1 | 4 | (Davis and Wilde 1996) |
| Paracetamol | 103-90-2 | N02BE01 | 3000 | 58.3 | 79.2 | 2 | 5 | (Escolà Casas et al. 2021; Wishart et al. 2018) |
| Propranolol | 318-98-9 | C07AA05 | 160 | 1.1 | 1.7 | 1.5 | 6 | (Escolà Casas et al. 2021) |
| Trimethoprim | 738-70-5 | J01EA01 | 400 | 0.4 | 0.4 | 40 | 52 | (Escolà Casas et al. 2021) |
| Venlafaxine | 93413-69-5 | N06AX16 | 100 | 7.5 | 12.3 | 5.3 | 5.9 | (Escolà Casas et al. 2021) |

# Results

## QC

A super QC were made by mixing aliquots of the samples. It was repeatedly injected for each 8 samples during the analysis of samples, to investigate the behaviors of the LC-QTOF system over time.

Quality control charts were constructed (Fig. S2, Fig. S3, and Fig. S4), by dividing the calculated concentration of a micropollutant with the average concentration of that micropollutant and then plotting all data as boxplot. Micropollutants with the large variation were often the ones close to LOQ, see an example of Valsartan in Fig. S3.


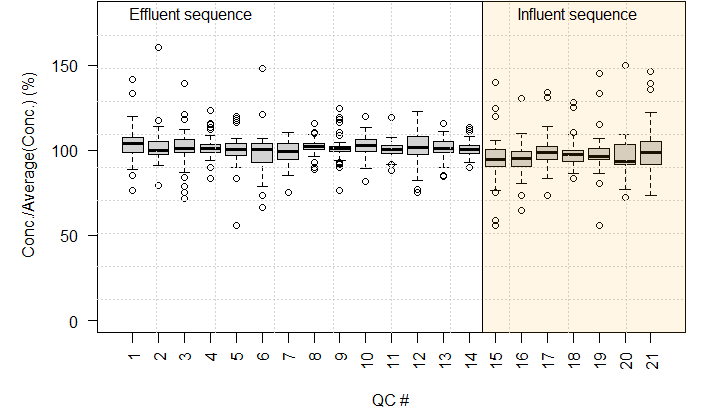


Fig. S2: Concentrations of the analytes measured in the super QC samples across the effluent and influent sequence for the LC-positive mode measured sequences.


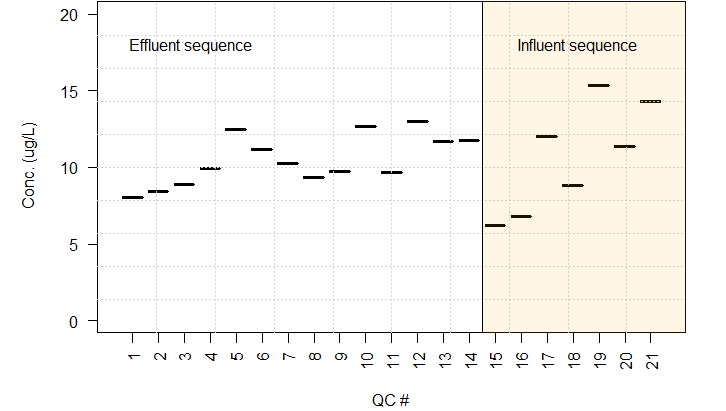


Fig. S3: Valsartan concentrations in superQC (not corrected for dilution), limit of quantification 5.25 ug/L (not corrected for dilution).


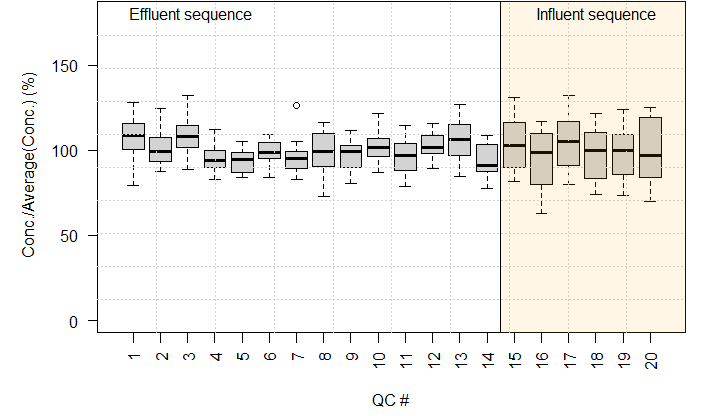


Fig. S4: Concentrations of the analytes measured in the super QC samples across the effluent and influent sequence for the LC negative mode measured sequences.

## Recovery experiment SPE

Recovery of MPs were investigated by spiking 100 µg L^-1^ MiliQ water, Tap water, and effluent wastewater prior to and after SPE. The samples were then injected together with non-spiked samples that was used for correction of analytes present in the matrix. The experiment was carried out as triplicates. The recoveries have been calculated and are presented in Table S6.

Table S6: The recovery calculated for investigated compounds. The recovery of a few selected MPs, the recoveries has been tested using MiliQ water, tap water and effluent wastewater.

| Compound | LogD (pH 7) | Log Kow | MilliQ Water (%) | Tap water (%) | Effluent wastewater (%) |
| --- | --- | --- | --- | --- | --- |
| 1,3-Diphenylguanidine | 2.46 | 2.96 | 88±3 | 84±3 | 78±9 |
| 1-Aminobenzotriazole | 0.55 | 1.45 | 59±5 | 52±8 | 70±6 |
| 1-Hydroxybenzotriazole | 0.07 | 0.69 | 98±6 | 85±2 | 189±29 |
| Amitriptyline | 2.96 | 0.86 | 75±11 | 63±6 | 69±10 |
| Atorvastatin | 1.25 | 6.81 | 28±31 | 7±22 | 71±12 |
| Carbamazepine | 0.29 | 2.45 | 103±4 | 90±4 | 109±12 |
| Cetirizine | -0.55 | 1.70 | 116±11 | 97±8 | 85±16 |
| 1-(4-Chlorophenyl)piperazine | 0.68 | 0.53 | 47±9 | 44±30 | 75±7 |
| Citalopram | 1.27 | 2.50 | 86±8 | 74±6 | 79±11 |
| Clarithromycin | 2.38 | 3.16 | 73±11 | 53±24 | 84±12 |
| Cyanoguanidine | -1.09 | -1.15 | 88±2 | 28±74 | 41±30 |
| DEET | 2.24 | 2.18 | 86±8 | 73±9 | 87±5 |
| Diatrizoic acid | -1 | 0.49 | 36±32 | 41±23 | 34±25 |
| Diuron | 0.13 | 2.68 | 93±7 | 74±9 | 93±7 |
| Gabapentin | -1.4 | -1.10 | 72±6 | 40±22 | 14±37 |
| Guanylurea | -1.82 | -3.57 | 65±15 | 66±16 | 88±9 |
| Ibuprofen | 0.45 | 3.74 | 73±20 | 85±12 | 95±23 |
| Isoproturon | 2.45 | 2.69 | 98±5 | 80±6 | 96±3 |
| Losartan | 1.29 | 3.47 | 81±9 | 72±1 | 62±18 |
| Metoprolol | -0.25 | 0.79 | 91±8 | 83±3 | 81±4 |
| Paracetamol | 0.4 | 0.46 | 89±5 | 59±26 | 93±7 |
| Sertraline | 3.14 | 4.81 | 78±9 | 70±3 | 71±7 |
| Sulfamethoxazol | -0.56 | 0.89 | 87±4 | 80±2 | 88±5 |
| Tebuconazole | 3.74 | 3.89 | 87±14 | 83±4 | 89±11 |
| Terbutryn | 1.38 | 3.56 | 86±6 | 71±8 | 83±12 |
| Valsartan | -0.89 | 0.90 | 92±8 | 78±6 | 89±5 |
| Venlafaxine | 1.43 | 3.13 | 93±6 | 79±4 | 94±6 |

## Investigation of fate of micropollutants through WWTP


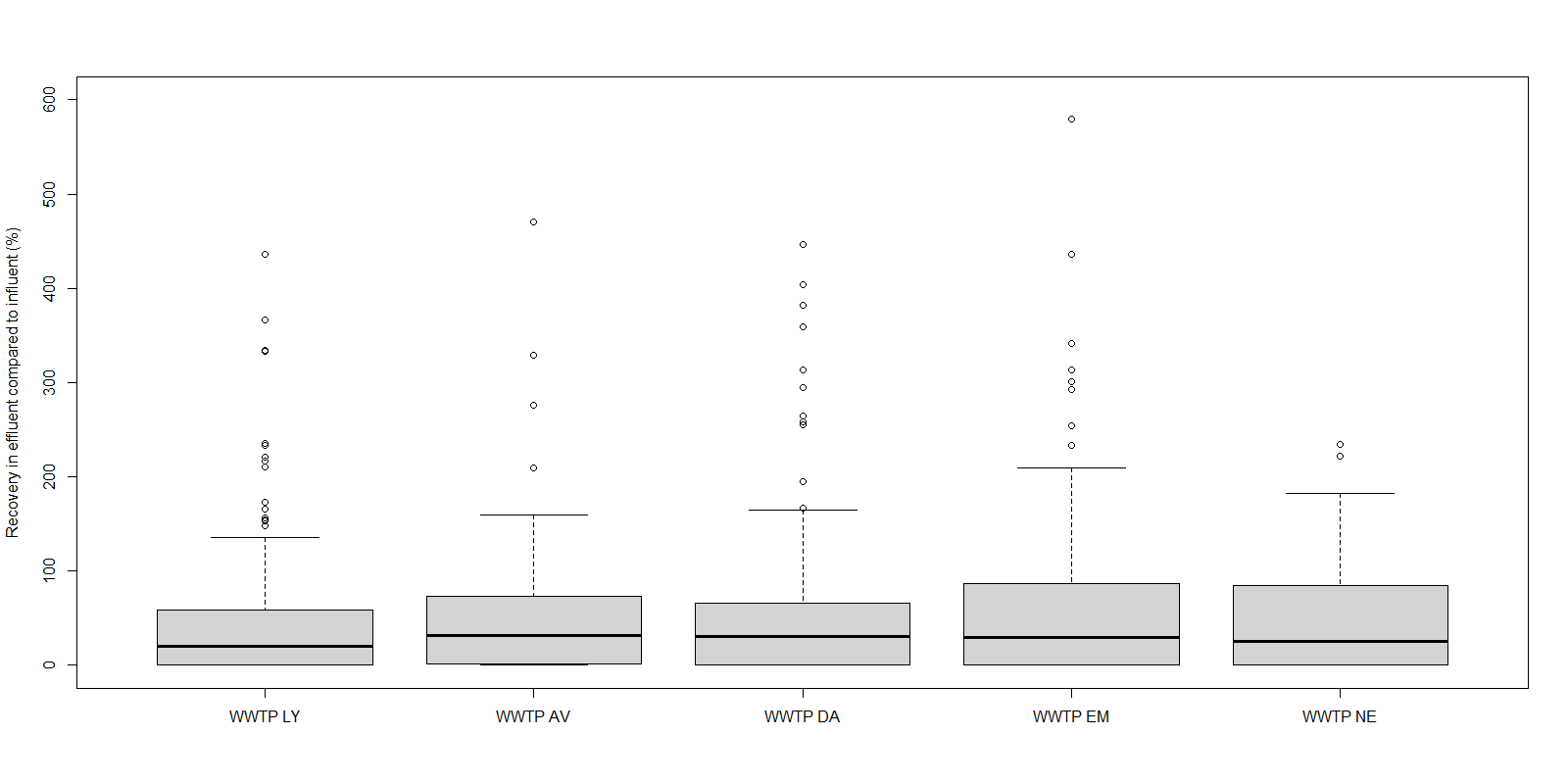


Fig. S5: Boxplot of all recoveries of the five investigated WWTPs. The average recoveries were found to be 39 %, 52 %, 62 %, 58 %, and 46 % for WWTP LY, WWTP, AV, WWTP DA, WWTP EM, and WWTP NE, respectively.

## Investigation of catchment areas


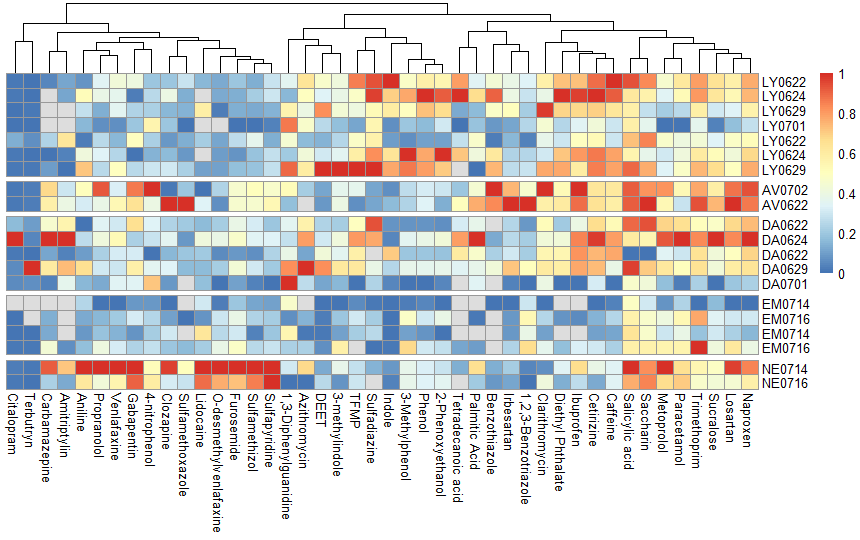


Fig. S6: Heatmap including hierarchical clustering of influent wastewaters for all micropollutants (with more than 12 quantifications). For each micropollutant the concentration has been range scaled (lowest value = 0 and highest value = 1).

# References

Aoshima, M., Fukasawa, K., & Kaneko, K. (2001). Absorption, distribution, metabolism and excretion of [14C]levocetirizine, the R enantiomer of cetirizine, in healthy volunteers. *European Journal of Clinical Pharmacology*, *57*(8), 571–582. https://doi.org/10.1007/s002280100364

Dalgaard, L., & Larsen, C. (1999). Metabolism and excretion of citalopram in man: identification of O-acyl- and N-glucuronides. *Xenobiotica; the Fate of Foreign Compounds in Biological Systems*, *29*(10), 1033–1041. https://doi.org/10.1080/004982599238092

Danmmarks Statistik. (2021). *statistikbanken.dk*. https://www.statistikbanken.dk

emc. (2022). *https://www.medicines.org.uk/emc/*. https://www.medicines.org.uk/emc/

Escolà Casas, M., Schröter, N. S., Zammit, I., Castaño-Trias, M., Rodriguez-Mozaz, S., Gago-Ferrero, P., & Corominas, Ll. (2021). Showcasing the potential of wastewater-based epidemiology to track pharmaceuticals consumption in cities: Comparison against prescription data collected at fine spatial resolution. *Environment International*, *150*, 106404. https://doi.org/10.1016/j.envint.2021.106404

FDA. (n.d.). *FDA Approved Drug Products: EQUETRO (carbamazepine) extended-release capsules*. www.fda.gov/medwatch

Hollender, J., Rothardt, J., Radny, D., Loos, M., Epting, J., Huggenberger, P., Borer, P., & Singer, H. (2018). Comprehensive micropollutant screening using LC-HRMS/MS at three riverbank filtration sites to assess natural attenuation and potential implications for human health. *Water Research X*, *1*. https://doi.org/10.1016/j.wroa.2018.100007

Luke, D. R., & Foulds, G. (1997). Disposition of oral azithromycin in humans. *Clinical Pharmacology and Therapeutics*, *61*(6), 641–648. https://doi.org/10.1016/S0009-9236(97)90098-9

Oyehaug, E., Ostensen, E. T., & Salvesen, B. (1984). High-performance liquid chromatographic determination of citalopram and four of its metabolites in plasma and urine samples from psychiatric patients. *Journal of Chromatography*, *308*, 199–208.

Portnoy, J. M., & Dinakar, C. (2004). Review of cetirizine hydrochloride for the treatment of allergic disorders. *Expert Opinion on Pharmacotherapy*, *5*(1), 125–135. https://doi.org/10.1517/14656566.5.1.125

Sheehan, J. J., Sliwa, J. K., Amatniek, J. C., Grinspan, A., & Canuso, C. M. (2010). Atypical Antipsychotic Metabolism and Excretion. *Current Drug Metabolism*, *11*(6), 516–525. https://doi.org/10.2174/138920010791636202

Sonje, V. M., Kumar, L., Meena, C. L., Kohli, G., Puri, V., Jain, R., Bansal, A. K., & Brittain, H. G. (2010). *Atorvastatin Calcium* (pp. 1–70). https://doi.org/10.1016/S1871-5125(10)35001-1

Tisler, S., Pattison, D. I., & Christensen, J. H. (2021). Correction of Matrix Effects for Reliable Non-target Screening LC–ESI–MS Analysis of Wastewater. *Analytical Chemistry*, *93*(24), 8432–8441. https://doi.org/10.1021/acs.analchem.1c00357

Wishart, D. S., Feunang, Y. D., Guo, A. C., Lo, E. J., Marcu, A., Grant, J. R., Sajed, T., Johnson, D., Li, C., Sayeeda, Z., Assempour, N., Iynkkaran, I., Liu, Y., Maciejewski, A., Gale, N., Wilson, A., Chin, L., Cummings, R., Le, D., … Wilson, M. (2018). DrugBank 5.0: a major update to the DrugBank database for 2018. *Nucleic Acids Research*, *46*(D1), D1074–D1082. https://doi.org/10.1093/nar/gkx1037
